# Pharmacological Activities and Safety of Ganoderma lucidum Spores: A Systematic Review

**DOI:** 10.7759/cureus.44574

**Published:** 2023-09-02

**Authors:** Nguyen Huu Lac Thuy, Vo Linh Tu, Le Nguyen Anh Thu, Tran Thanh Giang, Dao Tang Khanh Huyen, Duong Hoang Loc, Dao Ngoc Hien Tam, Nguyen Tuan Phat, Hong-Han Huynh, Thien Tan Tri Tai Truyen, Quang-Hien Nguyen, Uyen Do, Dang Nguyen, Truong Van Dat, Le Huu Nhat Minh

**Affiliations:** 1 Faculty of Pharmacy, University of Medicine and Pharmacy at Ho Chi Minh City, Ho Chi Minh City, VNM; 2 Faculty of Traditional Medicine, University of Medicine and Pharmacy at Ho Chi Minh City, Ho Chi Minh City, VNM; 3 Department of Chemistry and Biochemistry, University of Texas at Arlington, Arlington, USA; 4 Department of Regulatory Affairs, Asia Shine Trading & Service Company Ltd, Ho Chi Minh City, VNM; 5 Faculty of Medicine, Hue University of Medicine and Pharmacy, Hue, VNM; 6 Department of Cardiovascular Research, Methodist Hospital Southlake, Merrillville, USA; 7 International Master Program for Translational Science, College of Medical Science and Technology, Taipei Medical University, Taipei, TWN; 8 Faculty of Medicine, Nam Can Tho University, Can Tho City, VNM; 9 Science Department, Lone Star College, Houston, USA; 10 Department of Medical Engineering, University of South Florida, Tampa, USA; 11 Research Center for Artificial Intelligence in Medicine, Taipei Medical University, Taipei, TWN; 12 International Ph.D. Program in Medicine, College of Medicine, Taipei Medical University, Taipei, TWN

**Keywords:** sporoderm-broken extract, natural proteoglycan, antibacterial effect, ruizhi, biological activity, spore, reishi, lingzhi, ganoderma lucidum

## Abstract

*Ganoderma lucidum* is traditionally used to prevent and treat some diseases such as liver disorders, hypertension, insomnia, diabetes, and cancer. *G. lucidum* spore extracts are also reported to share similar bioactivities as extracts from its other parts. However, there is no systematic review that elucidates its pharmacological effect. Our aim is to comprehensively summarise current evidence of *G. lucidum* spore extracts to clarify its benefits to be applied in further studies. We searched five primary databases: PubMed, Virtual Health Library (VHL), Global Health Library (GHL), System for Information on Grey Literature in Europe (SIGLE), and Google Scholar on September 13, 2021. Articles were selected according to inclusion and exclusion criteria. A manual search was applied to find more relevant articles. Ninety studies that reported the pharmacological effects and/or safety of *G. lucidum* spores were included in this review. The review found that *G. lucidum* spore extracts showed quite similar effects as other parts of this medicinal plant including anti-tumor, anti-inflammatory, antioxidant effects, and immunomodulation. *G. lucidum* sporoderm-broken extract demonstrated higher efficiency than unbroken spore extract. *G. lucidum* extracts also showed their effects on some genes responsible for the body's metabolism, which implied the benefits in metabolic diseases. The safety of *G. lucidum* should be investigated in depth as high doses of the extract could increase levels of cancer antigen (CA)72-4, despite no harmful effect shown on body organs. Generally, there is a lot of potential in the studies of compounds with pharmacological effects and new treatments. Sporoderm breaking technique could contribute to the production of extracts with more effective prevention and treatment of diseases. High doses of *G. lucidum* spore extract should be used with caution as there was a concern about the increase in CA.

## Introduction and background

In the past, lingzhi has been known as a magic herb as well as an auspicious symbol by the Chinese. It is also known as "reishi," "shenzhi," and "xiancao," which mean good fortune and mysterious power. Taoism played an important role in promoting lingzhi for either medical purposes or otherwise. In the ancient era, people used the fruit body of *Ganoderma lucidum*, which has bioactive compounds, including sterols, triterpenoids, fatty acids, and carbohydrates. *G. lucidum* is traditionally used to prevent and treat some diseases such as liver disorders, hypertension, insomnia, diabetes, and cancer [1]. *G. lucidum* is known for its pharmacological activities that help promote human health [2]. 

*G. lucidum* spores are the fungus's mature germ cells, considered the essential and best part of the *G. lucidum* fruit body produced during the reproductive stage [3,4]. However, there are very few studies on *G. Lucidum *spore extract because the extracting procedure of the sporoderm is very difficult [5]. In recent years, thanks to spore-breaking techniques, the compounds inside *G. lucidum* spores have been studied more. *G. lucidum* spores have effects similar to the fruit body; moreover, their bioactive compounds, including sterols, triterpenoids, fatty acids, and carbohydrates show higher concentrations than other parts of this fungus [3,6]. Understanding the biological effects, dosages, uses, pharmacological mechanisms, and safety of* G. lucidum* spores will help increase the effectiveness of using* G. lucidum* spores as well as developing products from them. However, no systematic review has been reported on these data.

Therefore, in our study, we summarize the existing evidence to assess the biological activity and safety of G. lucidum spores and their compounds with the help of a systematic review.

## Review

Methods

Our systematic review followed the Preferred Reporting Items for Systematic Reviews and Meta-Analysis (PRISMA) checklist (Appendix 1) [7]. Our review protocol was registered at the International Prospective Register of Systematic Reviews (PROSPERO) (ID number CRD42021279806).

Eligibility Criteria

All types of original studies (in vitro, in vivo, clinical trial, case reports, retrospective study), published in English up to September 13, 2021, which provided information about the pharmacological effect and/or safety of *G. lucidum* (lingzhi or reishi) spores, as well as their compounds, were included. Articles that only reported the efficacy of* G. lucidum* fruit bodies, mycelia, or other species of *Ganoderma* but not *G. lucidum,* and studies with unreliable data (such as abstract-only articles, conference papers, theses, posters, editorials, and letters) were excluded.

Search Strategies

The search was performed on the following five databases: PubMed, Virtual Health Library (VHL), Global Health Library (GHL), System for Information on Grey Literature in Europe (SIGLE), and Google Scholar by search terms given in Table 1. To find other relevant research, a manual search was conducted utilizing the references of the included articles.

**Table 1:** Details of search terms in each database

	Databases	Search Terms	Results
1	PubMed	(“ganoderma lucidum” OR “G. lucidum” OR lingzhi OR reishi OR mannentake) AND (spore OR spores)	186
2	WHO Global Health Library (GHL)	(“ganoderma lucidum” OR “G. lucidum” OR lingzhi OR reishi OR mannentake) AND (spore OR spores)	31
3	Virtual Health Library (VHL)	(“ganoderma lucidum” OR “G. lucidum” OR lingzhi OR reishi OR mannentake) AND (spore OR spores)	181
4	Google Scholar	with all the words: spore with at least one of the words: "ganoderma lucidum" "G lucidum" lingzhi reishi mannentake in the title of article	261
5	SIGLE	“Ganoderma lucidum” OR “G. lucidum” OR lingzhi OR reishi OR mannentake	11

Study Selection and Data Collection

We used the WebPlotDigitizer tool at https://automeris.io/WebPlotDigitizer/ to extract data from the chart. The search results were automatically filtered for duplicate entries using Endnote X8.1 (Clarivate Plc, London, United Kingdom). Two independent reviewers selected articles based on title and abstract screening, followed by full-text screening. Any disagreements were resolved through discussion. Two independent reviewers extracted data from each article. The main data were the preparation methods of *G. lucidum* spores and their pharmacological activities. Data were grouped by pharmacological activity and study design.

Risk of Bias

The modified Consolidated Standards of Reporting Trials (CONSORT) checklist [8] was used for in vitro studies (Appendix 2). Regarding the introduction, all of the studies included a structured summary of the trial design, methods, results, conclusions establishing the scientific background, explanation of rationale, and the specific hypotheses to be examined. Randomization criteria (to assess sample standardization) and protocol criteria were not applied to assess study quality. A study with a score of 9-10 was considered "low risk of bias", 7-8 was considered "moderate risk of bias", 5-6 was considered "high risk of bias", and a score less than 5 was excluded from our systematic review.

In vivo studies were evaluated by the Systematic Review Centre for Laboratory Animal Experimentation (SYRCLE)ʼs tool (Appendix 3) [9]. A “yes” judgment indicated a low risk of bias, a “no” judgment indicated a high risk of bias, and the judgment was considered “unclear” if insufficient details have been reported to assess the risk of bias properly. Cohort studies and case reports were evaluated using the Study Quality Assessment Tools (SQAT) [10] of the National Institute of Health. Ratings for each item ranged from 0 for potential flaws to 1 for good practice (Appendices 4, 5). Additionally, we followed SQAT’s instructions to categorize "NA" (not applicable), "NR" (not reported), or "CD" (cannot determine). These were used for ambiguous fields when our investigators were not sure what score should be allotted, which suggested scientists should be cautious of potential flaws while adopting data from those studies. Each item received an equal number of points in the final percentage calculation. The scoring cut-off at 75% or above of the total points was considered "good" quality (low risk of bias), of which 75% and 43% were "fair" (moderate risk of bias), and articles that are 43% or below are considered "poor" quality (high risk of bias).

Clinical trials were evaluated using Risk of Bias 2 (RoB 2) from Cochrane (Appendices 6, 7) [11]. Ratings for each domain ranged from “low”, “some concerns” to “high”. A study that had all its domains rated "low" was considered "low risk of bias", if at least one domain was rated "some concerns" and none of them were "high", it was considered "some concerns" (moderate risk of bias), and if at least one domain is rated as "high" or the majority of domains are rated as "some concerns", it was considered "high risk of bias".

Results

**Figure 1:**
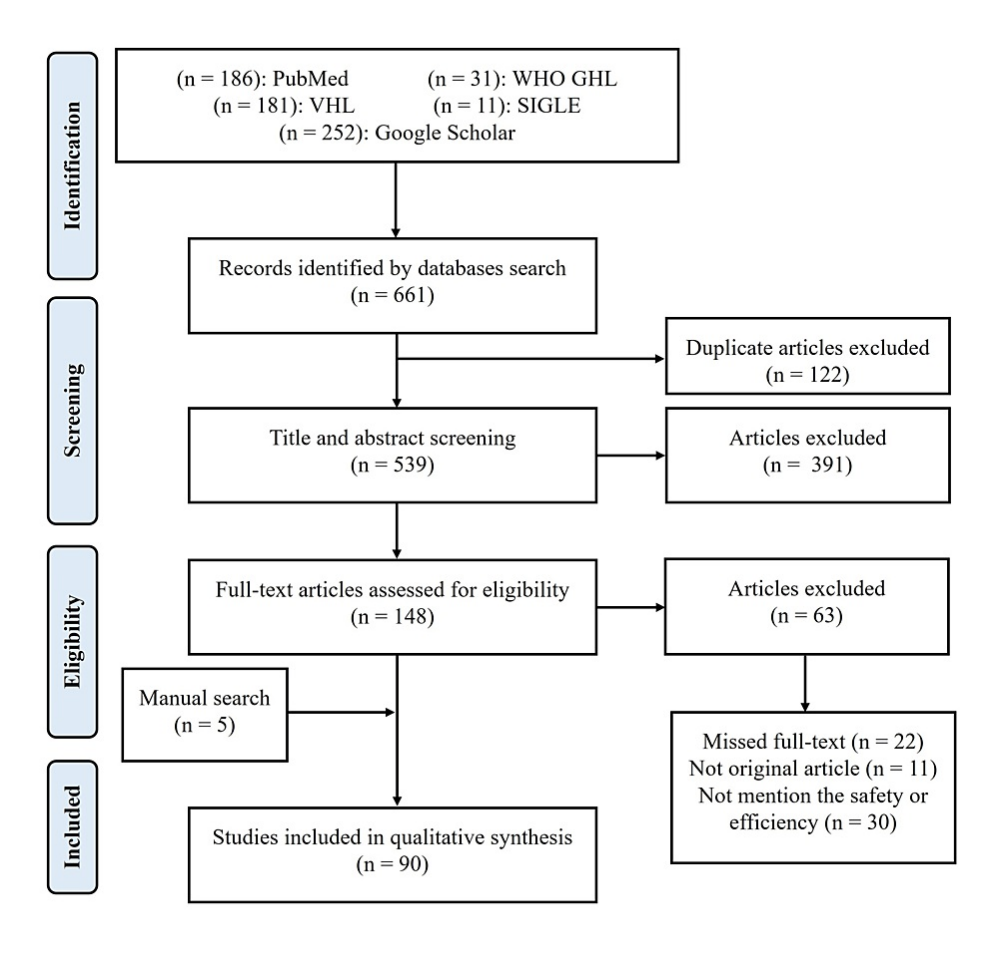
PRISMA flow diagram of study selection PRISMA: Preferred Reporting Items for Systematic Reviews and Meta-Analysis; WHO GHL: World Health Organization Global Health Library; SIGLE: System for Information on Grey Literature in Europe; VHL: Virtual Health Library

A total of 661 articles resulted from the database search. Of these, 122 were duplicates and excluded. The remaining 539 articles are screened and finally, 90 articles were included in the final analysis. The PRISMA flow diagram is presented in Figure 1. Among the included 90 articles, there were 40 in vitro studies, 26 in vivo studies, 18 studies that were both in vivo and in vitro, three clinical trials, two case reports, and one retrospective study.

 *Activities Against Cancer*

*G. lucidum* spores have a variety of activities in fighting against cancer. The long-chain fatty acids in ethanol extract from *G. lucidum* spores show cell proliferation inhibitory in vitro on HL-60 cells [12,13]. The ethanol extract of *G. lucidum* spores has a stronger inhibitory activity on HUC-PC and MCT-11 cells in vitro than the aqueous extract [14]. Alcohol extract of *G. lucidum* spores can inhibit human breast cancer cells (MDA-MB231) [15], non-small cell lung cancer (NCI-H460), colorectal adenocarcinoma (HCT-15) [16], and human leukemia THP-1 in vitro [17]. Triterpenoid extract from *G. lucidum* spores showed activities against cervical cancer Hela cells [18]. Spores of *G. lucidum* also suppress invasion of breast cancer MDA-MB-231 and prostate PC-3 cells by inhibiting transcription factors [19,20]. *G. lucidum* spore extract show antitumor-mediated and immunomodulatory ability to significantly reduce PD-1 protein in B lymphocytes [21].

Studies showed that sporoderm-broken spores of *G. lucidum* (BSG) show excellent fighting capacity against cancer in vitro and in vivo. In an experimental mouse, oral administration of BSG (2, 4, and 8 g/kg per day) was able to significantly impede the growth of sarcoma S180, hepatoma, and reticulocyte sarcoma L-II cells. Tumor weight was significantly reduced by 14.1, 18.,5, and 16.6% compared with the control group [22]. In mice models inoculated with 4T1-breast cancer, treatment with BSG (400 mg/kg) showed a significantly lower tumor weight compared with the control group (387 ± 23 mg vs. 512 ± 45 mg, p < 0.05) [23]. Water extract of BSG (BSGWE) was seen to inhibit many cancer cell lines in vitro such as human osteosarcoma (HOS, U2, MG63) [24,25], murine osteosarcoma (K7M2) [24], human colorectal cancer (HCT116, HT-29) [26,27], murine metastatic breast cancer (4T1) [23,28], murine sarcoma 180 (S180) [29], HeLa [30,31], human CCA TFK-1 [32], and hepatocellular carcinoma (H22) [33].

In in vivo study, treatment of 0.5 mg BSGWE for four weeks significantly reduced tumor weight and volume of K7M2 cells transplanted into mice [24]. In a mouse model inoculated with HOS stably transfected cells into the tibia, treatment with BSGWE 600 mg/kg for 21 days significantly reduced tumor weight and volume (p < 0.01) [25]. In a HCT116 xenograft mouse model, six weeks of oral treatment with BSGWE inhibited tumor growth, tumor volume was reduced by 23.8 (dose of 150 mg/kg) and 47.8% (dose of 300 mg/kg), respectively (p < 0.05). The final tumor weight at surgery at both doses was significantly lower compared with the control group; 1.27 ± 0.19 g (150 mg/kg) and 1.00 ± 0.21 g (300 mg/kg) (p < 0.05 for both), respectively, in comparision with 2.22 ± 0.11 g (control) and 1.28 ± 0.23 g (treated with 5-FU) [26]. In an HT-29 xenograft mouse model, treatment with polysaccharide extracted from BSG (BSGP) (300 mg/kg) significantly reduced tumor mass and volume compared with the control group [27]. BSGP showed significant inhibition of S180 and 4T1 breast cancer growth in mice. In a mouse model inoculated with S180 cancer cells, 14 days of treatment with BSGP (100 and 200 mg/kg) significantly reduced tumor weight compared with the control group (physiological saline) (p < 0.05 and p < 0.01); inhibitor ratio was 49.1 and 59.9%, respectively [29]. Treatment with BSGP (10 mg/kg, 30 mg/kg, 100 mg/kg) for 21 days resulted in tumor weights (0.84 ± 0.32 g, 0.82 ± 0.34 g, 0.86 ± 0.16 g, respectively) compared with 1.45 ± 0.24 g in the control group (p < 0.01), while the tumor weight in cyclophosphamide (CTX) -treated group (30 mg/kg) was 0.88 ± 0.40 g [34]. Moreover, BSGP (200 mg/kg and 400 mg/kg) showed excellent effect when the tumor weight was lower than the group treated with paclitaxel (PTX), and significantly lower compared with the control group (p < 0.05) [28].

Ethanol extracts of BSG (BSGEE) significantly inhibited HCT116 cell proliferation in vitro (p < 0.01) in nude mice through multiple mechanisms [35]. The mean weights of tumor were 0.86 ± 0.28 (model group), 0.59 ± 0.20 (75 mg/kg), and 0.38 ± 0.23 g (150 mg/kg) (p < 0.05) [35]. A study examining the anti-tumor activity of BSGEE and ethanol/aqueous extract of BSG (BSGEA) showed that BSGEE inhibited the growth of all three lung cancer cell lines (A549, H441, and H661) with an IC50 of 150 µg/ml while BSGEA did not show efficacy up to 1000 µg/ml [36]. In the xenograft mouse model with human lung cancer A549 cells, treatment with BSGEE (200 mg/kg per day) for four weeks showed a mean tumor volume reduction of 39.35% compared with the control group (p < 0.05). The average tumor weight was 0.90 g in BSGEE-treated mice compared with 1.54 g in control mice (p < 0.05) [36].

A study comparing the anti-tumor activity of BSG and *G. lucidum* sporoderm-nonbroken (NBSG) showed that the purity of BSG was more active than that of NBSG against cancer cells including SGC-7901, HeLa [37]. In a mouse model subcutaneously implanted with mouse S-180, treatment of 2 g/kg BSG and NBSG showed a 31.5% and 22.4% reduction in tumor weight, respectively, compared with untreated controls [38]. Two kinds of *G. lucidum* spore powder, BSG and sporoderm-removed *G. lucidum* (RSG) were compared in vivo andin vitro antitumor activities. The results showed that RSG exhibited stronger tumor suppressor activities than BSG in in vitro, and in the zebrafish model, the inhibition rate on gastric cancer cell SGC-7901, lung cancer cell A549, and B lymphocyte cell line Ramos of RSG was 78%, 31%, and 83%, respectively [39]. RSG also showed greater inhibition of three types of human gastric cancer cell lines (MKN28, AGS, NCI‑N87) than BSG [40].

*G. lucidum* oil, lipid substance extracted from the *G. lucidum* spore, also showed strong anti-tumor activity. In in vitro, *G. lucidum* oil inhibited human acute myeloid leukemia cell (HL-60), human chronic myeloid leukemia cell (K562), human gastric carcinoma cell (SGC7901) [41], human breast carcinoma cell (MDA-MB-231) [42], and miR-378M cell [43]. In in vivo, *G. lucidum* oil (1.2 g/kg) significantly suppressed the growth of murine sarcoma (S180) and murine hepatoma (H22) transplant tumors. The inhibitory rate was 30.9% (p < 0.05) and 44.9% (p < 0.01), respectively [41]. *G. lucidum *oil (6 g/kg) once daily orally in mice significantly reduced tumor volume of 4T1-breast cancer after 21 days (p < 0.05); there was no significantly different from PTX (10 mg/kg twice weekly) [42]. Notably, *G. lucidum* oil nanosystems showed better antitumor activity against human gastric cancer cells (MGC803) than *G. lucidum* oil, due to improved absorption efficiency and cell storage of *G. lucidum* oil nanosystems. In mice, treatment with *G. lucidum* oil 40 nm-nanosystems for 22 days reduced the tumor volume from 891 mm^3^ to 286 mm^3^, a therapeutic effect similar to CTX (40 mg/kg) [44].

Treatment with *G. lucidum* spore in gynecological cancer patients showed stable disease status in three out of six cases, while in the placebo group, all patients showed progressive disease [45]. Administration of *G. lucidum* spore twice daily in five cases of gastric cancer showed increased serum levels of tumor marker, CA72-4 [46]. A clinical study of 48 breast cancer patients showed that administration of *G. lucidum* spore powder (1000 mg three times daily) for four weeks resulted in significant improvements in areas of physical, reducing anxiety and improving the quality of life. Immune parameters such as tumor necrosis factor alpha (TNF-α) and interleukin-6 (IL-6) were also improved [47].

Immunomodulatory Activities of G. lucidum Spores

The polysaccharides of *G. lucidum* spores (SGP) were the most reported components of immunological activity. β-D-(1→3)-glucan SGP at concentrations of 1-100 µg/mL displayed a dose-dependent T lymphocyte-stimulating activity induced by concanavalin A [48]. The carboxymethylated derivatives of polysaccharides (1 or 100 µg/mL) also enhanced the proliferation of T and B lymphocyte, as it will be decreased as the level of substitution increased. Substitute compounds with lower levels seem to be more active than higher ones [49]. SGP showed a dose-dependent stimulation of lymphocyte proliferation in mice induced by concanavalin A and lipopolysaccharide [50].

*G. lucidum* mycelium extract induced human peripheral blood mononuclear cell (PBMC) and monocyte proliferation, while in contrast, *G. lucidum* spore extract suppressed PBMCs [51]. In addition, SGP significantly suppressed the proliferation of T cell in the association with increased IL-10 production [52]. For splenic mononuclear cells, treatment with SGP (at concentrations of 200, 400, and 800 mg/ml) significantly increased the proliferation of mononuclear cells and increased cytokine production (IL-2, TNF-α) [53]. In another study, microwave-treated SGP also significantly stimulated the secretion of cytokine production (TNF-α, IL-6) [54]. Extracts of *G. lucidum* spores (40 mg/ml and 80 mg/ml) significantly enhanced the function of human polymorphonuclear neutrophils (PMNs) (both p < 0.05). Extracts of *G. lucidum* spores may have modulated human immunity through the p38 mitogen-activated protein kinase pathway [55].

The immunological activity of *G. lucidum* spores has also been tested in animals. Especially, β-D-glucan as an immunostimulator has attracted much attention because it is beneficial for the treatment of cancers. β-D-(1→3)-glucan (dose of 25 or 50 mg/kg) for four successive days in mice showed an enhancing effect on T lymphocyte proliferation, significantly different from the control group [48]. The carboxymethylated α-D-(1→3)-glucan (dose of 25 or 50 mg/kg) also substantially enhanced the proliferation of T and B lymphocyte [49]. The native glucan, named PGL (doses of 25 mg/kg and 50mg/kg) had a strong effect on suppressing the antibody production in mice (p < 0.05). And the effect at a higher dose of 50 mg/kg was stronger than that at a lower dose of 25 mg/kg [56]. The degraded glucan showed a greater ability to increase T and B lymphocyte proliferation and production of antibodies against sheep red blood cells in mice than native glucan [57]. Intraperitoneal treatment of SGP (dose of 50, 100, 200 mg/kg) for 10 days significantly increased the concanavalin A-induced proliferative response of splenocytes. In addition, two-week transperitoneal SGP showed dose-dependent inhibitory activities on tumor growth of Lewis lung cancer in C57BL/6 mice [54].

Crude SGP and refined SGP have shown activity in the immune system of BALB/c mice. Crude polysaccharide and refined polysaccharide treatment for 30 days suppressed mitogen-induced splenocyte proliferation (concanavalin A or lipopolysaccharide) (p < 0.05). Interestingly, tumor-killing ability of NK cells was significantly promoted by crude polysaccharides (p < 0.01) but not refined polysaccharides while only refined polysaccharides promoted the activation of T cells [58]. Meanwhile, GLSB70 and GLSB50, two polysaccharide fractions obtained from aqueous extracts of NBSG can stimulate humoral immunity in mice immunosuppressed with CTX. GLSB50 and GLSB70 (300 mg/kg per day) showed extremely significant increases in HC50 values (serum half-hemolytic values) (p < 0.01 and 0.05, respectively). GLSB50 exhibited better and comparable activity to the positive control lentinan [59]. In another study, NK cell cytotoxicity and macrophage phagocytosis were also significantly enhanced by the lipid fraction, and G. lucidum oil (800 mg/kg).* G. lucidum* oil showed immune-enhancing effects on both innate and cellular immunity and significantly increased the intestinal Bacteroidetes/Firmicutes ratio [60].

BSG and RSG showed immunological activity in the zebrafish model as significantly improved neutrophils (p < 0.05 or 0.01) after 24 h, RSG exhibited greater activity. Moreover, only RSG was able to significantly promote macrophage formation (p < 0.01) [61]. In mice, β-glucan from BSG (dose of 75, 150, 300 mg/kg) could promote dinitrochlorobenzene to delayed ear swelling similar lentinan (150 mg/kg) [62]. CTX-induced immune suppression and SGP can counteract CTX toxicity and restore the immune system. In mice treated with SGP (50 mg/kg/day) thymus weight was significantly higher than in mice treated with CTX alone (p < 0.05) [63].

A randomized controlled double-blind trial in postoperative patients with breast and lung cancer showed that treatment with *G. lucidum* spore powder (2000 mg, twice daily for six weeks) increased CD3+ CD4+ CD3+ HLADR- cell types, whereas decreased CD4+ CD25+ Treg, CD3+ HLADR+ cell types compared to control [64].

Anti-inflammatory of G. lucidum Spores

In vitro study that simulates digestion has shown that RSG can promote the release of the active ingredient more readily than other forms of *G. lucidum* spores so that the active ingredients are more easily absorbed. In particular, BSGWE has the best anti-inflammatory effect on the intestines [65].

BSGP significantly reduced the expressions of pro-inflammatory cytokines in mice fed with a high-fat diet. BSGP also had gut microbiota modulating activities (increased *Allobaculum, Bifidobacterium, *and decreased *Lachnospiraceae_UCG-001, Ruminiclostrdium*) [66]. Besides, pretreatment with a high dose of *G. lucidum* spores (1 g/kg per day) can relieve symptoms of sialoadenitis in non-obese diabetic mice [67].

Antioxygenation Activity of G. lucidum Spores and Reduction of Oxidative Stress

The radical scavenging activity of *G. lucidum* spore increased as the concentration increased. The percentage inhibition of 1,1-diphenyl-2-picrylhydrazyl (DPPH)radical of triterpenoids was 62.16% at 400 µg/ml [68]. In another study, the percentage inhibition of DPPH radical of triterpenoids (600 μg/ml) reached a maximum (61.09 ± 1.38%) [18]. A novel natural proteoglycan from BSG and NBSG also showed antioxidant activity with DPPH scavenging activity of 90.6 ± 8.5% and 72.6 ± 3.7%, and with ABTS scavenging effect of 73.3 ± 6.7% and 47.2 ± 5.9%, respectively [31].

The breaking techniques and extraction solvent for *G. lucidum* spores may affect free radical scavenging activity. Among the reported methods, maceration with spheres of various materials extract contained the most significant antioxidant activity, with 57.22 ± 0.09% [69]. Phenolic and polysaccharide extracts also showed different antioxidant capacities [70].

In the reducing power assay, *G. lucidum *spore powder revealed high antioxidant activity, the reducing power of* G. lucidum* spore powder increased with an increase in drying temperature (from 95°C to 105°C), in some cases even higher than the antioxidant property of ascorbic acid [71].

In a rabbit ischemia/reperfusion (I/R) model, pretreatment with BSG was shown to minimize damage, inhibiting the negative effects of I/R on both response compliance. That mean BSG can reduce oxidative stress [72]. In the *Drosophila melanogaster* model, the* G. lucidum* oil-treated groups had mean and maximum lifespans significantly longer than untreated groups, under both normal and oxidative stress conditions.* G. lucidum *oil treatment markedly affected the activity of antioxidant enzymes such as increasing total superoxide dismutase and catalase activities and decreasing malondialdehyde levels [73].

Protective Activity of G. lucidum Spores

Studies showed that *G. lucidum* spores or extracts of *G. lucidum* spores have protective capabilities such as retinal protection [74], cardiac protection [75-77], hepatic protection [78], intestinal protection [79], neuroprotective effect [80], bone marrow cells protection [81] and efficiency on apoptosis [74,79,82].

Organ protection against apoptosis by pre-treatment with *G. lucidum* spores has been observed in in vivo studies. Pre-treatment with *G. lucidum* spores (50, 100, 150 mg/mL, for 19 days) showed a dose-dependent reduction in the splenic index and significantly different apoptosis compared with the model group (p < 0.05) [82]. *G. lucidum* spore lipid administration inhibited N-methyl-N-nitrosourea-induced retinal photoreceptor apoptosis in vivo (p < 0.01 on days 1 and 3) [74]. SGP shows promising protective activities against PTX-induced small intestinal barrier injury by inhibiting apoptosis, and promoting small intestinal cells’ proliferation [79].

Pre-treated* G. lucidum* spore oil (5mL, @P188/PEG400) nanosystem four to eight hours before X-ray irradiation protected H9C2 cells from X-rays (16 Gy) (cell viability of H9C2 cells increased to 101.4-112.3%. Moreover, treatment with G. lucidum spore oil (5mL, @P188/PEG400) nanosystem in mice significantly reduced X-ray-induced necrosis [75]. *G. lucidum* extracts also increased heart function [76,77].

In a mice model of cadmium chloride (CdCl2)-induced hepatotoxicity (3.7 mg Cd (II)/kg, i.p.), seven days of pre-treatment with *G. lucidum* spore reduced liver enzymes (Alanine transaminase (ALT), aspartate aminotransferase (AST)) and liver weight/body weight ratio [78]. In the nervous system, pre-treatment with a high dose of *G. lucidum* spores (8 g/kg) was shown to help protect neurons from apoptosis, and ameliorate cognitive dysfunction in rats undergoing intracerebroventricular injection of streptozotocin procedure [80]. In vivo trials in mice showed that *G. lucidum* spores could protect bone marrow mesenchymal stem cell and promote hematopoiesis recovery in CTX-treated [81].

Antimicrobial Activities of G. lucidum Spore

The aqueous extract of *G. lucidum* spore had antibacterial properties against *Staphylococcus aureus*, *Escherichia coli*, *Enterococcus faecalis*, and *Klebsiella pneumoniae* (minimal inhibitory concentration (MIC) of 125 mcg/ml, 125 mcg /ml, less than 02 mcg/ml, and 62.5 mcg/ml, respectively [83]. The Mann‐Whitney U test and Chi‐square test showed that there was no significant difference between the antibacterial effect of mycelium and spores against P. intermedia and that both mycelium and spores were effective (MIC of 5.64 and 3.62 mcg/ml, respectively [84]. Besides, topical application of *G. lucidum* spore powder or aqueous or organic solvents also showed antibacterial effects [85].

The antibacterial effect against *S. aureus*, *E. coli* was also tested with different extracts from *G. lucidum* spores. The extracted triterpenoids showed that the diameter of the inhibition zone for both bacteria was significant [18]. Chitosan from *G. lucidum* spore powder obtained through both thermal deoxidation, (TCD) and emerging ultrasonic-assisted deoxidation (USAD) also displayed enhancement of antibacterial zone against both *E. coli and S. aureus*, USAD extraction showed higher activity [86]. A novel natural proteoglycan from cracked (proteoglycan-C) and uncracked *G. lucidum* spore powder (proteoglycan-UC) also showed activity against these two bacteria [31].

The antibacterial activity of BSG and spores lipid was tested in a mice model against infection with *Mycobacterium tuberculosis*. The mean bacterial load at week 24 was approximately 2.5 log10 CFU in the lungs, and more than 4 log10 CFU in the spleen, showing significant statistical difference compared to the control group [87].

Metabolism and G. lucidum Spore

*G. lucidum *spore and its extraction are considered to be potential in hypoglycemic and hypolipidemic activities. These activities were presented by blood glucose level [88-90], glycated hemoglobin (HbA1c) [89] and blood total cholesterol (TC), triglyceride (TG) and high-density lipoprotein cholesterol (HDL-C) levels [78,88-91].

In glycemic metabolism, in vitro studies show that *G. lucidum* spore powder extracts such as triterpenoids or proteoglycan can modulate insulin sensitivity in insulin-resistant HepG2 cells and reduce glucose concentration [31,68]; moreover, oligosaccharide of *G. lucidum* spore can be considered to use as an effective prebiotic [92]. In in vivo studies, treatment with resistant starch spores (10.5 g/kg bw/day) in diabetic rats reduced blood glucose level by 21.9% in week 3, and it was also significantly lower than the model group (p < 0.05) [88]. In the streptozotocin (STZ)-induced diabetic rats model, there was a significant reduction in blood glucose in the *G. lucidum* spores group compared with the STZ group (23.98 ± 5.20 mmol/L vs 30.08 ± 3.13 mmol/L, p < 0.05). HbA1c decreased by 6% in the *G. lucidum* spores group compared with the STZ group (but no significant difference) [89]. Treatment of *G. lucidum* spore powder in diabetic rats for four weeks also decreased blood glucose levels (p < 0.05). Blood glucose levels in the intervention group and model group were 24.31 ± 1.17 mmol/L and 32.22 ± 1.71 mmol/L, respectively [90]. In addition, by the effect of *G. lucidum* spore and BSGEE [91] or SGP [89,90]), the HDL-C value in the intervention group increased [88,91], and reduced serum level of TG, TC, and LDL-C [89,91]. Moreover,* G. lucidum* spore powder significantly inhibited body weight from increasing under a high-fat diet. *G. lucidum* spore powder may tend to reduce serum TG while it had no effects on HDL [66].

Efficiency on Alzheimer’s Disease

In the Morris water maze, RSG (360 and 720 mg/kg) ameliorated amyloid β (Aβ) deposition and Tau phosphorylation, and prevented the reductions of neurotrophin brain-derived neurotrophic factor (BDNF) and tropomyosin-related kinase B receptor in the hippocampus in sporadic Alzheimer’s disease rats. Therefore, BSG enhanced memory and showed potential for the prevention and treatment of Alzheimer’s disease [93].

Wound-healing Activity of G. lucidum Spore

Skin wound healing assay performed on mice showed using *G. lucidum* oil increased collagen deposition in skin burn injury. Moreover, *G. lucidum* oil significantly accelerated skin wound healing and reduced levels of inflammatory cytokines [94].

Induction of Proliferator-activated Receptor Alpha Activity

Based on fold induction data, it is found that *G. lucidum* spore lipid potently and selectively induced the activity of PPARα. As a result, *G. lucidum* spore lipid may be the potential the in treatment of many diseases such as hyperlipidemia, modulating the immune reaction specifically, suppressing chronic inflammation [95].

Proliferation Enhancers

*Ganoderma* spores extract at 0.01% and 0.1% (wt/vol) significantly promoted embryonic stem cell growth (p < 0.05) [96].

Epilepsy Treatment

In vitro experiments showed the antiepileptic activity of *G. lucidum *spore. The expression of NT-4 in *G. lucidum* spore group was higher than model group (p > 0.01), and at 0.122 mg/ml concentration *G. lucidum* spore for best effects [97]. Ganoderic acids from* G. lucidum *also showed antiepileptic potential based on the evaluation of apoptosis, and BDNF and TRPC3 expression, especially at 80 μg/ml [98]. A retrospective study of 18 patients with epilepsy showed that using* G. lucidum* spore reduced the weekly seizure frequency from 3.1 ± 0.8 to 2.4 ± 1.2 (p = 0.04) [99].

Anti-aging Activity of G. lucidum Spore

The anti-aging effect of ganodermasides A and ganodermasides B from *G. lucidum* spores was shown through upregulation of UTH1 expression and extending the replicative life span of yeast [100].

The pharmacological activities of *G. lucidum* spore are listed in Table 2.

**Table 2:** Pharmacological activities of Ganodema lucidum spore ^# ^mean ± SEM (standard error of the mean) GLS: *G. lucidum* spore; GLSAE: *G. lucidum* spore alcohol extract; SB: sporoderm broken; GLSP: *G. lucidum* spore polysaccharide; MTT: 3-[4,5-dimethylthiazol-2-yl]-2,5 diphenyl tetrazolium bromide; BSG: sporoderm‑broken spores of *G. lucidum*; BSGP: sporoderm‑broken spores of *G. lucidum* polysaccharide; RSGP: polysaccharide of sporoderm‑removed spores of *G. lucidum; *TGF: transforming growth factor; E/E-BSG: Ethanol/ethnol extrct of sporoderm‑broken spores of *G. lucidum*; GLSO@NE: *G. lucidum* spore oil nanosystems; PARP: poly (ADP-ribose) polymerase; GLSO: *G. lucidum* spore oil; BSGEE: ethanol extract of sporoderm‑broken spores of *G. lucidum*; BSGWE: water extract of sporoderm‑broken spores of *G. lucidum; *BGLSP: wall-broken *G. lucidum* spore powder; RGLSP: wall-removed *G. lucidum* spore powder; PTX: paclitaxel; GLP: *G. lucidum* polysaccharide; CRC: colorectal cancer; PBMC: peripheral blood mononuclear cells; GL-M: *G. lucidum* mycelium extract; GL-SG: pure spore polysaccharides; TNF: tumor necrosis factor; GSG: *G. lucidum* spores glucan; PMN: polymorphonuclear neutrophil; LPS: lipopolysaccharide; CGLP: crude polysaccharide of *G. lucidum; *DPPH: 1,1-diphenyl-2-picrylhydrazyl; WGLP: water-soluble polysaccharide derived from *G. lucidum* spores; SBGS: sporoderm-broken germinating spores; LNT: lentinan; RPGS: refined polysaccharides of *G. lucidum *spores; GLSW: water soluble β-glucan; EGLS: encapsulated GLS within resistant starch; EEG: GLS ethanol extract; STZ: streptozotocin

Author (Year)	Pharmacological activities	Intervention/ Control	Dose	Result (Mean ± SD)	Conclusion
in vitro					
Fukuzawa et al., (2008) [12]	Antitumor effect	Spore extract	100 μg/ml	HL-60 growth = 117.35 ± 19.56 (% of control) ^(*)^	GLS could cause HL-60 cells to enter an early apoptosis
			150 μg/ml	HL-60 growth = 97.79 ± 12.35 (% of control) ^(*)^	
			200 μg/ml	HL-60 growth = 61.76 ± 35 (% of control) ^(*)^	
			250 μg/ml	HL-60 growth = 23.68 ± 24.7 (% of control) ^(*)^	
			300 μg/ml	HL-60 growth = 4.12 ± 4.12 (% of control) ^(*)^	
		Control		HL-60 growth = 100 (% of control) ^(*)^	
Xinlin et al., (1997) [37]	Antitumor effect	GLSAE-SB	1000 µg/ml	OD value (Hela cell) = 0.186 ± 0.00038 (p < 0.01 vs. control) OD value (HepG2 cell) = 0.172 ± 0.0058 (p < 0.01 vs. control) OD value (SGC-7901 cell) = 0.201 ± 0.0021 (p < 0.01 vs. control) OD value (HL60 cell) = 0.286 ± 0.005 (p < 0.01 vs. control) OD value (L1210 cell) = 0.487 ± 0.0045 (p < 0.01 vs. control)	GLS was able to inhibit cancer cell lines such as Hela, HepG2, SGC-7901, HL60, and L1210
		Control		OD value (Hela cell) = 0.356 ± 0.0046 OD value (HepG2 cell) = 0.342 ± 0.0052 OD value (SGC-7901 cell) = 0.561 ± 0.0053 OD value (HL60 cell) = 0.365 ± 0.0049 OD value (L1210 cell) = 0.53 ± 0.0048	
Lu et al., (2004) [14]	Antitumor effect	Spore ethanol extract		IC_50_ (HUC-PC cells) = 280 µg/ml IC_50_ (MTC-11 cells) = 234 µg/ml	When compared to water extracts, ethanol extracts demonstrated a greater growth-inhibiting impact
		Spore water extract		IC_50_ (HUC-PC cells) = 500 µg/ml IC_50_ (MTC-11 cells) = 465 µg/ml	
Lu et al., (2004) [15]	Antitumor effect	Ethyl acetate fraction	40 μg/ml	Proliferation human umbilical vein endothelial cell = 50.92 ± 10.5 (%) (p < 0.05 vs. control) ^(*)^ Proliferation breast cancer MDA-MB231 cell = 26.31 ± 5.26 (%) ^(*)^	The alcohol extract of GLS has anti-breast cancer effects by anti-proliferative of tumor cells and endothelial cells
		Control		Proliferation human umbilical vein endothelial cell = 100 ± 27.53 (%) ^(*)^ Proliferation breast cancer MDA-MB231 cell = 100 ± 42.30 (%) ^(*)^	
Oliveira et al., (2014) [16]	Antitumor effect	Spore methanol extract		GI_50_ (NCI-H460 cells) = 386.9 ± 11.15 µg/ml GI_50_ (HCT-15 cells) = 280.8 ± 11.17 µg/ml	Methanolic spore extracts are considered highly effective against tumors
Sliva et al., (2002) [19]	Antitumor effect	GLS	0mg/ml	Migration (MDA-MB-231cells) = 97.71 ± 11.29 (%) ^(*)^ Migration (PC-3 cells) = 100 ± 14 (%) ^(*)^ Relative NF-kB activity = 100.34 ± 13.296 (%) ^(*)^ Relative AP-1 activity = 101.04 ± 9.10 (%) ^(*)^	GLS inhibited breast cancer cell motility in a dose-dependent manner
			0.5 mg/ml	Migration (MDA-MB-231cells) = 84.73 ± 6.87 (%) ^(*)^ Migration (PC-3 cells) = 63.7 ± 8.07 (%) ^(*)^ Relative NF-kB activity = 85.64 ± 9.115 (%) ^(*)^ Relative AP-1 activity = 68.53 ± 5.596 (%) ^(*)^	
			1.2 mg/ml	Migration (MDA-MB-231cells) = 22.9 ± 14.1 (%) ^(*)^ Migration (PC-3 cells) = 39.51 ± 7.26 (%) ^(*)^ Relative NF-kB activity = 73.77 ± 9.796 (%) ^(*)^ Relative AP-1 activity = 57.34 ± 6.65 (%) ^(*)^	
			2.5 mg/ml	Migration (MDA-MB-231cells) = 12.21 ± 4.58 (%) ^(*)^ Migration (PC-3 cells) = 16.12 ± 2.42 (%) ^(*)^ Relative NF-kB activity = 67.83 ± 0.70 (%) ^(*)^ Relative AP-1 activity = 46.15 ± 3.496 (%) ^(*)^	
Sliva et al., (2003) [20]	Antitumor effect	Whole spores	2.5 mg/ml	Migration (MDA-MB-231cells) = 12.923 ± 1.385 (%) ^(*)^ NF-kB activity (%) = 29 ± 4.6 (%) (p < 0.005) ^(*)^ Migration (PC-3 cells) = 16.154 ± 2.769 (%) ^(*)^ NF-kB activity (%) = 35 ± 14.5 (%) (p < 0.005) ^(*)^	Strong anti-cancer activity of GLS has been demonstrated against breast and prostate cancer cells
		Broken spores	2.5 mg/ml	Migration (MDA-MB-231cells) = 28.615 ± 4.154 (%) ^(*)^ NF-kB activity (%) = 29 ± 0.8 (%) ^(*)^ Migration (PC-3 cells) = 6 ± 0.462 (%) ^(*)^ NF-kB activity (%) = 2 ± 0.2 (%) (p < 0.05) ^(*)^	
		Control	0 mg/ml	Migration (MDA-MB-231cells) = 99.231 ± 12 (%) ^(*)^ NF-kB activity (%) = 100 ± 5.7 (%) (p < 0.05) ^(*)^ Migration (PC-3 cells) = 98.769 ± 10.616 (%) ^(*)^ NF-kB activity (%) = 100 ± 7.6 (%) ^(*)^	
Song et al., (2021) [33]	Antitumor effect	GLSP + primary macrophages (Mø)	400 μg/ml	The inhibiton rate (H22 cells) = 18.4 ± 1.8 (%) (p < 0.01 vs control) ^(*)^	The MTT experiment demonstrated that GLSP+Mø significantly and dose-dependently reduced the growth of H22 cells
			800 μg/ml	The inhibiton rate (H22 cells) = 27.8 ± 1.8 (%) (p < 0.01 vs control) ^(*)^	
		Control	0 μg/ml	The inhibiton rate (H22 cells) = 0 (%)	
Wang et al., (2019) [21]	Mediated immunomodulation and cancer treatment	GLS extract	0.5 mg/ml	Fold change in PD -1 protein = 0.38 ± 0.01 Fold change in PD -1 protein = 1.71 ± 0.01 % of PD-1 cells = 1.8 ± 0.01 (%) Fold change in CCL5 protein = 12.63 ± 2.73 (p < 0.5) Fold change in CCL5 protein = 35.37 ± 3.28 (p < 0.1)	*G. lucidum* could be used to develop novel immunomodulators to prevent and treat cancer along with many other illnesses
		Control		Fold change in PD -1 protein = 0.92 ± 0.01 Fold change in PD -1 protein = 1.17 ± 0.01 % of PD-1 cells = 3.7 ± 0.01 (%) Fold change in CCL5 protein = 1.05 ± 0.01 Fold change in CCL5 protein = 0.89 ± 0.01	
Zhong et al., (2021) [40]	Antitumor effect	BSGP		IC_50_ (MKN28 cells) = 18.88 ± 1.58 (mg/ml) IC_50_ (NCI‐N87 cells) = 13.44 ± 0.73 (mg/ml) IC_50_ (AGS cells) = 11.76 ± 1.16 (mg/ml)	RSGP may be a promising autophagy inhibitor in the treatment of gastric cancer as it is more effective than BSGP at reducing gastric cancer cell viability
		RSGP		IC_50_ (MKN28 cells) = 5.03 ± 1.62 (mg/ml) IC_50_ (NCI‐N87 cells) = 8.08 ± 1.39 (mg/ml) IC_50_ (AGS cells) = 3.76 ± 2.85 (mg/ml)	
Zhu et al., (2000) [30]	Antitumor effect	Extract I (SB)		IC_50_ (HeLa cells) = 4.46 (mg/ml)	It was discovered that extracts I and III from spores with fractured sporoderm inhibited cell proliferation in a dose-dependent way
		Extract I subjected to silica gel chromatography (Extract III)		IC_50_ (HeLa cells) = 0.75 (mg/ml)	
Wu et al., (2012) [43]	Antitumor effect	Ganoderma	0.4 µl/ml	Cell number (miR-378) = 136.36 ± 6.06 (%) ^(*)^	The miR-378 cells' sensitivity to epirubicin was considerably boosted by the addition of *Ganoderma* oil
		Epirubicin	2 µg/ml	Cell number (miR-378) = 88.25 ± 10.23 (%) (p < 0.01 vs control) ^(*)^	
		*Ganoderma* + Epirubicin	0.4 µl/ml + 2 µg/ml	Cell number (miR-378) = 28.03 ± 4.16 (%) (p < 0.01 vs. control) ^(*)^	
Li et al., (2016) [32]	Inhibits cholangiocarcinoma cell migration	TGF-β1	2 ng/ml	Number of cell migration = 170.9 ± 15.28 ^(*)^	TFK-1 cells' TGF-1-induced migration was prevented by the GLS extract
		TGF-β1 + GLE	2 ng/ml + 400μg/ml	Number of cell migration = 48.72 ± 7.28 (p < 0.01 versus TGF-β1 alone) ^(*)^	
		TGF-β1 + GLE	2 ng/ml + 800μg/ml	Number of cell migration = 36.36 ± 8.73 (p < 0.01 versus TGF-β1 alone) ^(*)^	
		Control (DMSO)		Number of cell migration = 21.81 ± 6.55 (p < 0.01 versus TGF-β1 alone) ^(*)^	
Chen et al., (2016) [41]	Antitumor effect	Ganoderma spores oil		IC_50_ (K562 cells) = 1.13 mg/mL IC_50_ (K562 cells) = 2.27 mg/mL IC_50_ (K562 cells) = 6.29 mg/mL	GBS oil caused dose-dependent cytotoxicity in K562, HL60 and SGC-7901 cells
Chen et al., (2016) [36]	Antitumor effect	E/E-BSG	100 μg/ml	Migration (H441 cells) = 81.02 ± 1.5 (% of control) (p < 0.05 vs control)^ (*)^	Lung cancer cell viability and migration were significantly inhibited by oily extracts of BSG
			200 μg/ml	Migration (H441 cells) = 63.18 ± 3.8 (% of control) (p < 0.01 vs control)^ (*)^	
			300 μg/ml	Migration (H441 cells) = 17.83 ± 4.6 (% of control) (p < 0.001 vs control)^ (*)^	
		Negative control (0 μg/ml)		Migration (H441 cells) = 100 ± 3.0 (% of control)^ (*)^	
		E/E-BSG	10 μg/ml	Colony number (A549 cells) = 67.26 ± 6.12 (% of control) (p < 0.05 vs control)^ (*)^	
		E/E-SBGS	50 μg/ml	Colony number (A549 cells) = 2.29 ± 1.53 (%of control) (p < 0.001 vs control)^ (*)^	
		Negative control (0 μg/ml)		Colony number (A549 cells) = 100 ± 10 (% of control)^ (*)^	
Dai et al., (2021) [44]	Antitumor effect	40 nm-GLSO@NEs		IC_50 _(MGC803) = 0.15 ± 0.01 (μl/ml)	The anticancer efficacy of various-sized GLSO@NEs was strong, and there was no evident toxicity
		40 nm-GLSO@NEs	0.1 μl/ml	Early apoptotic cells (MGC803 cells) = 0 ± 0.91 (%) ^(*)^ Late apoptotic cells (MGC803 cells) = 5.04 ± 1.37 (%) ^(*)^ Migrated cell (MGC803 cells) = 76.27 ± 13.98 (%) (p < 0.01 vs. control) ^(*)^ Invaded cell (MGC803 cells) = 88.24 ± 2.51 (%) (p < 0.01 vs. control) ^(*)^	
			0.2 μl/ml	Early apoptotic cells (MGC803 cells) = 9.62 ± 0.91 (%) (p < 0.05 vs. control) ^(*)^ Late apoptotic cells (MGC803 cells) = 36.18 ± 4.13 (%) (p < 0.01 vs. control) ^(*)^ Migrated cell (MGC803 cells) = 45.76 ± 8.9 (%) (p < 0.01 vs. control) ^(*)^ Invaded cell (MGC803 cells) = 52.94 ± 5.04 (%) (p < 0.01 vs. control) ^(*)^	
			0.4 μl/ml	Early apoptotic cells (MGC803 cells) = 28.85 ± 1.84 (%) (p < 0.01 vs. control) ^(*)^ Late apoptotic cells (MGC803 cells) = 39.39 ± 3.66 (%) (p < 0.01 vs. control) ^(*)^ Migrated cell (MGC803 cells) = 17.79 ± 5.09 (%) (p < 0.001 vs. control) ^(*)^ Invaded cell (MGC803 cells) = 23.98 ± 0.02 (%) (p < 0.001 vs. control) ^(*)^	
		Control		Early apoptotic cells (MGC803 cells) = 0 (%) ^(*)^ Late apoptotic cells (MGC803 cells) = 2.75 ± 0.91 (%) ^(*)^ Migrated cell (MGC803 cells) = 100 ± 4.24 (%) ^(*)^ Invaded cell (MGC803 cells) = 100 ± 3.36 (%) ^(*)^	
Jiao et al., (2020) [42]	Antitumor effect	Model		Fold change of control (PARP) = 1.02 ± 0.14 Fold change of control (caspase-3) = 1.02 ± 0.21	In MDA-MB-231 cells, GLSO upregulated the expression of Bax and caspase-3
		GLSO	0.2 µl/ml	Fold change of control (PARP) = 0.32 ± 0.01 (p < 0.001 vs. model) Fold change of control (caspase-3) = 1.12 ± 0.14	
			0.4 µl/ml	Fold change of control (PARP) = 0.28 ± 0.01 (p < 0.001 vs. model) Fold change of control (caspase-3) = 2.13 ± 0.1 (p < 0.001 vs. model)	
			0.6 µl/ml	Fold change of control (PARP) = 0.226 ± 0.01 (p < 0.001 vs. model) Fold change of control (caspase-3) = 3.45 ± 0.3 (p < 0.001 vs. model)	
Li et al., (2017) [34]	Antitumor effect	BSGEE	0 mg/ml	Cell viability = 100 (% of control) Cell cycle distribution (G0/G1) = 52.6 (%) Apoptosis = 10.37 (%) Average migration cells = 143.48 ± 15.21	HCT116 cell growth was significantly lowered by BSGEE in a dose- and time-dependent manner
			0.64 mg/ml	Cell viability (24h) = 93.75 ± 10.93 (% of control) Cell viability (48h) = 90.63 ± 6.24 (% of control) Cell viability (72h) = 75 ± 8.59 (% of control) (p < 0.05 vs. control)	
			1.6 mg/ml	Cell viability (24h) = 64.06 ± 10.94 (% of control) (p < 0.01 vs. control) Cell viability (48h) = 50 ± 6.25 (% of control) (p < 0.01 vs. control) Cell viability (72h) = 41.4 ± 2.35 (% of control) (p < 0.01 vs. control) Cell cycle distribution (G0/G1) = 56.62 (%) Apoptosis = 18.15 ± 2.59 (%) Average migration cells = 113.04	
			4 mg/ml	Cell viability (24h) = 25.78 ± 6.25 (% of control) (p < 0.01 vs. control) Cell viability (48h) = 10.15 ± 0.78 (% of control) (p < 0.01 vs. control) Cell viability (72h) = 6.25 ± 1.56 (% of control) (p < 0.01 vs. control) Cell cycle distribution (G0/G1) = 56.98 (%) Apoptosis = 21.48 ± 2.59 (%) Average migration cells = 50 ± 6.5	
			10 mg/ml	Cell viability (24h) = 14.84 ± 2.34 (% of control) (p < 0.01 vs. control) Cell viability (48h) = 8.59 ± 1.57 (% of control) (p < 0.01 vs. control) Cell viability (72h) = 3.91 ± 2.34 (% of control) (p < 0.01 vs. control) Apoptosis = 27 ± 2.63 (%) Average migration cells = 23.91 ± 6.52	
Na et al., (2017) [26]	Antitumor effect	Control	0 mg/ml	% cell viability = 100 ± 0.5 (% of control)	Colorectal cancer HCT116 cell viability was significantly lowered by BSGWE in a time- and dose-dependent manner
		BSGWE	1.25 mg/ml	% cell viability (24h) = 80 ± 0.5 (% of control) (p < 0.01 vs. control)	
			2.5 mg/ml	% cell viability (24h) = 75 ± 0.5 (% of control) (p < 0.001 vs. control)	
			5 mg/ml	% cell viability (24h) = 70 ± 0.5 (% of control) (p < 0.001 vs. control)	
			7.5 mg/ml	% cell viability (24h) = 68 ± 1 (% of control) (p < 0.001 vs. control)	
Shi et al., (2021) [39]	Antitumor effect	RGLSP		IC_50_ (SGC-7901 cells) = 1.9 (mg/mL) IC_50_ (A549 cells) = 2.526 (mg/mL)	The three tumor cell lines were inhibited by BGLSP and RGLSP in a dose-dependent manner
		BGLSP		IC_50_ (SGC-7901 cell) = 9.774 (mg/mL) IC_50_ (A549 cells) = 7.923 (mg/mL)	
Su et al., (2018) [23]	Antitumor effect	ESG	0 mcg/ml	Viability (24h) = 99.5 ± 1.5 (%) Viability (48h) = 98.74 (%)	GLS extract (12.5-200 μg/mL) treatments for 24 or 48 hours had no effect on the viability of 4T1 cells, suggesting that the anticancer activity of GLS extract was not directly mediated via cytotoxicity
			12.5 mcg.ml	Viability (24h) = 77.2 ± 4.68 (%) Viability (48h) = 93.46 (%)	
			25mcg/ml	Viability (24h) = 85.71 ± 3.83 (%) Viability (48h) = 87.43 (%)	
			50mcg/ml	Viability (24h) = 82.65 ± 4.59 (%) Viability (48h) = 91.59 (%)	
			100 mcg/ml	Viability (24h) = 79.59 ± 3.82 (%) Viability (48h) = 92.71 (%)	
			200 mcg/ml	Viability (24h) = 85.71 ± 6.36 (%) Viability (48h) = 84.42 (%)	
		Model		PD-1 mRNA relative fold of change in tumor = 1.42 ± 0.26 PD-1 µg/mg protein = 3.33 ± 0.33 CTLA-4 mRNA relative fold of change in tumor = 1.37 ± 0.29 CTLA-4 IOD/10^6^ pixel in tumor = 666 ± 166	
		ESGH	400 mg/kg	PD-1 mRNA relative fold of change in tumor = 0.71 ± 0.08 (p < 0.05 vs. model group) PD-1 µg/mg protein = 1.67 ± 0.083 (p < 0.01 vs. model group) CTLA-4 mRNA relative fold of change in tumor = 0.63 ± 0.1 (p < 0.05 vs model group) CTLA-4 IOD/10^6^ pixel in tumor = 1066 ± 300	
		ESGL	200 mg/kg	PD-1 mRNA relative fold of change in tumor = 1.45 ± 0.13 PD-1 µg/mg protein = 2.16 ± 0.167 (p < 0.01 vs. model group) CTLA-4 mRNA relative fold of change in tumor = 0.92 ± 0.08 (p < 0.05 vs model group) CTLA-4 IOD/10^6^ pixel in tumor = 400 ± 66.67	
Su et al., (2018) [28]	Antitumor effect	Model		IOD/10^6^ pixel = 5066 ± 2800	PTX and GLSP in combination showed greater tumor control
		SLP	200 mg/kg	IOD/10^6^ pixel = 800 ± 533	
		SHP	400 mg/kg	IOD/10^6^ pixel = 533 ± 400	
Zhang et al., (2019) [25]	Antitumor effect	BSGWE	2 mg/ml	HOS cell viability (24h) =125.84 (%) HOS cell viability (48h) = 100.42 (%) HOS cell viability (72h) = 76.27 (%) U2 cell viability (24h) = 81.36 (%) U2 cell viability (48h) = 87.71 (%) U2 cell viability (72h) =106.78 (%) MG63 cell viability (24h) = 102.96 (%) MG63 cell viability (48h) =110.59 (%) MG63 cell viability (72h) = 81.36 (%) HOS cell number = 312.33 ± 21.25 (%) U2 cell number = 482 ± 23.37 (%)	Osteosarcoma cell cycle progression at the G2/M phase was halted by BSGWE, which inhibited osteosarcoma cell proliferation and migration in a dose-dependent manner
			4 mg/ml	HOS cell viability (24h) = 67.37 (%) HOS cell viability (48h) = 40.67 (%) HOS cell viability (72h) = 10.17 (%) U2 cell viability (24h) = 66.1 (%) U2 cell viability (48h) = 50.84 (%) U2 cell viability (72h) = 44.49 (%) MG63 cell viability (24h) =30.51 (%) MG63 cell viability (48h) =15.25 (%) MG63 cell viability (72h) =24.15 (%) HOS cell cycle distribution (G2/M phase) =16.5 ± 0.82 (%) U2 cell cycle distribution (G2/M phase) = 14.98 ± 1.12 (%) HOS cell cycle distribution (G2/M phase) = 16.5 ± 0.82 (%) U2 cell cycle distribution (G2/M phase) = 14.98 ± 1.12 (%) HOS cell number = 180.67 ± 15.33 (%) U2 cell number = 124.67 ± 19.01 (%) Apoptotic cells = 23.69 ± 0.71 (%) Apoptotic cells = 8.86 ± 0.42 (%)	
			8 mg/ml	HOS cell cycle distribution (G2/M phase) =22.78 ± 0.73 (%) U2 cell cycle distribution (G2/M phase) = 21.23 ± 0.82 (%) HOS cell cycle distribution (G2/M phase) = 22.78 ± 0.73 (%) U2 cell cycle distribution (G2/M phase) = 21.23 ± 0.82 (%) Apoptotic cells = 62.8 ± 1.93 (%) Apoptotic cells = 32.14 ± 2.2 (%)	
		NC		HOS cell cycle distribution (G2/M phase) =11.42 ± 1.02 (%) U2 cell cycle distribution (G2/M phase) =8.9 ± 0.47 (%) HOS cell number = 498.67 ± 20.95 (%) U2 cell cycle distribution (G2/M phase) = 8.9 ± 0.47 (%) HOS cell number = 498.67 ± 20.95 (%) U2 cell number = 713.33 ± 27.08 (%)	
		Control		Apoptotic cells = 18.41 ± 2.97 (%) Apoptotic cells = 8.08 ± 0.27 (%)	
Pan et al., (2019) [27]	Antitumor effect	GLP	0	Cell viability 24h = 98.75 ± 5	GLP induced apoptosis of CRC cells
			2.5 mg/ml	Cell viability 24h = 71.86 ± 2.5	
			5 mg/ml	Cell viability 24h = 63.75 ± 3.13	
			10 mg/ml	Cell viability 24h = 48.75 ± 3.75	
Wang et al., (2012) [29]	Immunological activity, antitumor effect	RMPI-1640	0	Inhibitory ratio (Sarcoma 180 cells) = 0 (%) Inhibitory ratio (PG cells) = 0 (%)	BSGP did not inhibit the growth of S180 cells and PG cells
		BSGP	100 mg/l	Inhibitory ratio (Sarcoma 180 cells) = 3.3 (%) Inhibitory ratio (PG cells) = 2.0 (%)	
			400 mg/l	Inhibitory ratio (Sarcoma 180 cells) = 7.1 (%) Inhibitory ratio (PG cells) = 0.8 (%)	
He et al., (2020) [24]	Immunological activity, antitumor effect	NC		Early apoptosis rate (HOS) = 4.41 ± 1.18 (%) Late apoptosis rate (HOS) = 5.29 ± 1.47 (%)	BSGWE-induced osteosarcoma cell apoptosis
		BSGWE	2 mg/ml	Early apoptosis rate (HOS) = 10.59 ± 2.06 (%) (p < 0.001 vs. control) Late apoptosis rate (HOS) = 9.71 ± 1.47 (%) (p < 0.001 vs. control)	
			5 mg/ml	Early apoptosis rate (HOS) = 21.76 ± 3.53 (%) (p < 0.001 vs. control) Late apoptosis rate (HOS) = 10.29 ± 2.06 (%) (p < 0.001 vs. control)	
Bao et al., (2002) [48]	Immunological activity	PSGL-I-1A	1 µg/ml	A570 = 0.71 ± 0.03 (p < 0.05 vs. control)	At doses of 1-100 g/mL, the native glucan significantly increased T lymphocyte proliferation
			10 µg/ml	A570 = 0.85 ± 0.02 (p < 0.01 vs. control)	
			100 µg/ml	A570 = 0.89 ± 0.01 (p < 0.001 vs control)	
		Control	0 µg/ml	A570 = 0.64 ± 0.03	
Bao et al., (2001) [49]	Immunological activity	PSG-CM-1	1 µg/ml	A570 (T cell) = 0.65 ± 0.02 (p < 0.01 vs. control) A570 (B cell) = 0.54 ± 0.02 (p < 0.01 vs. control)	The carboxymethylated derivatives promote the growth of T and B lymphocytes
			100 µg/ml	A570 (T cell) = 0.75 ± 0.03 (p < 0.001 vs control) A570 (B cell) = 0.65 ± 0.03 (p < 0.001 vs control)	
		PSG-CM-2	1 µg/ml	A570 (T cell) = 0.62 ± 0.03 (p < 0.05 vs. control) A570 (B cell) = 0.49 ± 0.02 (p < 0.05 vs. control)	
			100 µg/ml	A570 (T cell) = 0.66 ± 0.02 (p < 0.01 vs. control) A570 (B cell) = 0.54 ± 0.01 (p < 0.01 vs. control)	
		PSG-CM-3	1 µg/ml	A570 (T cell) = 0.57 ± 0.04 A570 (B cell) = 0.44 ± 0.05	
			100 µg/ml	A570 (T cell) = 0.61 ± 0.03 (p < 0.05 vs. control) A570 (B cell) = 0.5 ± 0.02 (p < 0.05 vs. control)	
		Control	0 µg/ml	A570 (T cell) = 0.55 ± 0.03 A570 (B cell) = 0.41 ± 0.05	
Chan et al., (2005) [51]	Immunological activity	GLS extract	1mcg/mL	Relative cell proliferation (%) = 68.36 ± 10.21 (%) (p < 0.001 vs. control) ^(*)^	PBMCs and monocytes proliferated when exposed to GL-M, but GLS extract had a slight inhibitory impact
			10mcg/mL	Relative cell proliferation (%) = 70.4 ± 8.17 (%) (p < 0.001 vs. control) ^(*)^	
			100mcg/mL	Relative cell proliferation (%) = 69.38 ± 8.17 (%) (p < 0.001 vs. control) ^(*)^	
			1000mcg/mL	Relative cell proliferation (%) = 72.4489 ± 7.14 (%) (p < 0.001 vs. control) ^(*)^	
		GL-M	1mcg/mL	Relative cell proliferation (%) = 120.41 ± 8.16 (%) ^(*)^	
			10mcg/mL	Relative cell proliferation (%) = 148.97 ± 12.25 (%) (p < 0.01 vs. control) ^(*)^	
			100mcg/mL	Relative cell proliferation (%) = 153.06 ± 10.2 (%) (p < 0.01 vs. control) ^(*)^	
			1000mcg/mL	Relative cell proliferation (%) = 266.32 ± 27.55 (%) (p < 0.001 vs. control) ^(*)^	
		Negative control		Relative cell proliferation (%) = 100 (%) ^(*)^	
Chan et al., (2007) [52]	Immunological activity	GLS extract		Relative cell proliferation (%) = 69.3 ± 14.4 (%) (p < 0.001 vs. control) ^(*)^ IL-10 = 212.7 ± 121.5 (pg/mL) (p < 0.01 vs. control) ^(*)^	There was a significant suppression of T cell proliferation from the GLS extract-treated DC:T mixed lymphocyte reaction
		GL-SG		Relative cell proliferation (%) = 98.6 ± 14.3 (%) ^(*)^ IL-10 = 858.7 ± 182.3 (pg/mL) (p < 0.05 vs. control) ^(*)^	
		Negative control (RPMI)		Relative cell proliferation (%) = 100 (%) ^(*)^	
Guo et al., (2009) [54]	Immunological activity, antitumor effect	Unstimulated cells		TNF-α = 14.47 ± 13 (pg/ml) IL-6 = 111.47 ± 33 (pg/ml)	GSG could stimulate the MAPKs signal pathway and cause the production of TNF- and IL-6
		GLSP	50 μg/ml	TNF-α = 144.38 ± 19 (pg/ml) (p < 0.05 vs. control) IL-6 = 449.18 ± 42 (pg/ml) (p < 0.05 vs. control)	
			100 μg/ml	TNF-α = 251.87 ± 31 (pg/ml) (p < 0.05 vs. control) IL-6 = 731.14 ± 82 (pg/ml) (p < 0.05 vs. control)	
			200 μg/ml	TNF-α = 444.38 ± 37 (pg/ml) (p < 0.05 vs. control) IL-6 = 1032.78 ± 138 (pg/ml) (p < 0.05 vs. control)	
		GSG + PMB		TNF-α = 441.17 ± 24 (pg/ml) (p < 0.05 vs. control) IL-6 = 1013.11 ± 101 (pg/ml) (p < 0.05 vs. control)	
Yue et al., (2008) [38]	Immunological activity, antitumor effect	*Ganoderma* spore	1 g/kg	Proliferative respone = 4346.82 (%)	When compared to the pileus extract, BSG had higher growth-inhibiting properties
			2 g/kg	Proliferative respone = 6612.71 (%)	
			4 g/kg	Proliferative respone = 4670.52 (%)	
		Control		Proliferative respone = 3560.69 (%)	
Hsu et al., (2012) [55]	Immunological activity	G. lucidum spores extract	0 mg/ml	Phagocytic activity of PMNs = 42.92 ± 10.25 (%) (p < 0.05 vs. control) Phagocytic activity of PMNs with p38 MAPK inhibitor = 42.88 ± 19.06 (%) (p < 0.05 vs. control)	The p38 MAPK pathway is activated by the G. lucidum extract, which then modifies human immunity by stimulating human PMNs
			40 mg/ml	Phagocytic activity of PMNs = 54.02 ± 16.875 (%) (p < 0.05 vs. control) Phagocytic activity of PMNs with p38 MAPK inhibitor = 50.07 ± 6.705 (%) (p < 0.05 vs. control) Activation ratio = 0.496 ± 0.687 (p < 0.05 vs. control)	
			80 mg/ml	Phagocytic activity of PMNs = 57.22 ± 12.27 (%) (p < 0.05 vs. control) Phagocytic activity of PMNs with p38 MAPK inhibitor = 54.12 ± 11.79 (%) (p < 0.05 vs. control) Activation ratio = 0.506 ± 0.746 (p < 0.05 vs. control)	
			100 mg/ml	Phagocytic activity of PMNs = 59.16 ± 8.9 (%) (p < 0.05 vs. control) Phagocytic activity of PMNs with p38 MAPK inhibitor = 48.15 ± 9.67 (%) (p < 0.05 vs. control)	
Ma et al., (2008) [53]	Immunological activity	GLSP	0	Cell proliferation = 1 ± 0.05 (fold of control) ^(*)^ IL-2 production = 1.1 ± 0.03 (fold of control) ^(*)^ TNF-α production = 1 ± 0.2 (fold of control) ^(*)^	GLSP significantly enhanced IL-2 and TNF-production
			200 μg/ml	Cell proliferation = 1 ± 0.05 (fold of control) (p < 0.05 vs. control) ^(*)^ IL-2 production = 1.8 ± 0.02 (fold of control) (p < 0.05 vs. control) ^(*)^ TNF-α production = 3.4 ± 0.2 (fold of control) (p < 0.05 vs. control) ^(*)^	
			400 μg/ml	Cell proliferation = 1 ± 0.04 (fold of control) (p < 0.05 vs. control) ^(*)^ IL-2 production = 2.8 ± 0.25 (fold of control) (p < 0.05 vs. control) ^(*)^ TNF-α production = 4.8 ± 0.29 (fold of control) (p < 0.01 vs. control) ^(*)^	
			800 μg/ml	Cell proliferation = 1.3 ± 0.07 (fold of control) (p < 0.05 vs. control) ^(*)^ IL-2 production = 4.5 ± 0.19 (fold of control) (p < 0.01 vs. control) ^(*)^ TNF-α production = 5.6 ± 0.23 (fold of control) (p < 0.01 vs. control) ^(*)^	
Zhang et al., (2011) [50]	Immunological activity	LPS		A570 = 0.5 ± 0.02 (nm) ^(*)^	GLP might enhance the proliferation of lymphocytes stimulated by ConA or LPS
		ConA		A570 = 0.6 ± 0.04 (nm) ^(*)^	
		LPS+CGLP	50 µg/ml	A570 = 0.55 ± 0.12 (nm) ^(*)^	
			100 µg/ml	A570 = 0.62 ± 0.05 (nm) (p < 0.05 vs. control) ^(*)^	
		LPS+GLP	50 µg/ml	A570 = 0.61 ± 0.04 (nm) ^(*)^	
			100 µg/ml	A570 = 0.65 ± 0.05 (nm) p < 0.05 vs. control) ^(*)^	
		ConA+CGLP	50 µg/ml	A570 = 0.75 ± 0.02 (nm) (p < 0.01 vs. control) ^(*)^	
			100 µg/ml	A570 = 0.789 ± 0.001 (nm) (p < 0.01 vs. control) ^(*)^	
		ConA+GLP	50 µg/ml	A570 = 0.78 ± 0.08 (nm) (p < 0.01 vs. control) ^(*)^	
			100 µg/ml	A570 = 0.87 ± 0.03 (nm) (p < 0.01 vs. control) ^(*)^	
Cai et al., (2021) [65]	Anti-inflammatory	Water extract group	0.8 g	Indicator A = 0.64 ± 0.08 (mg/mL) ^(*)^ Indicator B = 0.18 ± 0.03 (mg/mL) (p < 0.05 vs. control) ^(*)^	The intestinal anti-inflammatory activities were better in the water extract than they were in the alcohol extract
		Alcohol extract group	0.8 g	Indicator A = 0.72 ± 0.06 (mg/mL) (p < 0.05 vs. control) ^(*)^ Indicator B = 0.13 ± 0.02 (mg/mL) (p < 0.05 vs. control) ^(*)^	
		Glucose control group	0.8 g	Indicator A = 0.57 ± 0.08 (mg/mL) (p < 0.05 vs. control) ^(*)^ Indicator B = 0.15 ± 0.01 (mg/mL) (p < 0.05 vs. control) ^(*)^	
Saavedra Plazas et al., (2020) [69]	Antioxidant activity	RM	1g	% inhibition DPPH = 47.85 ± 0.07 (%) ^AB^	BR extract had higher antioxidant activity
		BR	1g	% inhibition DPPH = 57.22 ± 0.09 (%) ^B^	
		MBR1	1g	% inhibition DPPH = 45.13 ± 0.03 (%) ^A^	
		Control (Unbroken spores)	1g	% inhibition DPPH = 46.83 ± 0.08 (%) ^AB^	
Dai et al., (2019) [75]	Protection against radiation-induced heart disease	GLSO@P188/PEG400 NS	0.5 μL/mL	Cell viability 0.5h = 94.43 ± 4.89 (% of control)^ (*)^ Cell viability 4h = 101.77 ± 8.15 (% of control)^ (*)^ Cell viability 8h = 112.36 ± 3.67 (% of control)^ (*)^	H9C2 cells were effectively protected against X-rays (16 Gy) by pre-treating GLSO@P188/PEG400 NS before IR for 4–8 hours
		Control		Cell viability = 100 (% of control)^ (*)^	
		X-ray alone (16 Gy)		Cell viability = 70.2 ± 7.9 (% of control)^ (*)^	
Nguyen and Nguyen (2015) [71]	Antioxidant activity	GLS powder	10 mg/ml	Antioxidant activity (95°C) = 1.32 ± 0.19 Antioxidant activity (100°C) = 2.14 ± 0.19 Antioxidant activity (105°C) = 2.66 ± 0.08 Antioxidant activity (AA°C) = 2.27 ± 0.06	The dried wall-broken spore powder had a strong antioxidant activity
			15 mg/ml	Antioxidant activity (95°C) = 2.48 ± 0.19 Antioxidant activity (100°C) = 2.93 ± 0.1 Antioxidant activity (105°C) = 3.06 ± 0.15 Antioxidant activity (AA°C) = 2.7 ± 0.04	
			20 mg/ml	Antioxidant activity (95°C) = 3.07 ± 0.25 Antioxidant activity (100°C) = 3.7 ± 0.18 Antioxidant activity (105°C) = 3.67 ± 0.11 Antioxidant activity (AA°C) = 2.81 ± 0.06	
Shen et al., (2019) [68]	Type 2 diabetes, mild DPPH radical scavenging activity, and inhibition of antioxidant activity	GLSP	10 µg/ml	DPPH radical-scavenging activities = 21.91 ± 1.39 (%) ^(*)^	Triterpenoid extract with good biocompatibility showed potential use for type 2 diabetes, mild DPPH radical scavenging activity, and inhibition of antioxidant activity
			50 µg/ml	DPPH radical-scavenging activities = 20.86 ± 7.66 (%) ^(*)^	
			100 µg/ml	DPPH radical-scavenging activities = 25.04 ± 7.3 (%) ^(*)^	
			200 µg/ml	DPPH radical-scavenging activities = 39.99 ± 3.23 (%) ^(*)^	
			300 µg/ml	DPPH radical-scavenging activities = 45.91 ± 8.35 (%) ^(*)^	
			400 µg/ml	DPPH radical-scavenging activities = 65.39 ± 3.82 (%) ^(*)^	
		Control		Glucose consumption = 6.47 ± 0.63 (mmol/L) ^(*)^	
		Metformin	0.001 mol/l	Glucose consumption = 1.21 ± 0.52 (mmol/L) ^(*)^	
		Triterpenoid	0.015 mg/ml	Glucose consumption = 0.94 ± 0.42 (mmol/L) ^(*)^	
			0.03 mg/ml	Glucose consumption = 1.1 ± 0.37 (mmol/L) ^(*)^	
			0.06 mg/ml	Glucose consumption = 2.53 ± 0.73 (mmol/L) (p < 0.01 vs. control) ^(*)^	
		Control		Glucose consumption = 0.83 ± 0.83 (mmol/L) ^(*)^	
		Insulin	5x10^-7^ mol/l	Glucose consumption = 1.06 ± 0.22 (mmol/L) ^(*)^	
		Metformin	0.001 mol/l	Glucose consumption = 2.29 ± 0.18 (mmol/L) (p < 0.01 vs. control) ^(*)^	
		Triterpenoid	0.015 mg/ml	Glucose consumption = 1.35 ± 0.06 (mmol/L) (p < 0.01 vs. control) ^(*)^	
			0.03 mg/ml	Glucose consumption = 1.82 ± 0.12 (mmol/L) (p < 0.01 vs. control) ^(*)^	
			0.06 mg/ml	Glucose consumption = 2.21 ± 0.28 (mmol/L) (p < 0.01 vs. control) ^(*)^	
Heleno et al., (2012) [70]	Antioxidant activity	FB-Ph		DPPH scavenging activity = 0.14 ± 0.01 (mg/ml) Reducing power = 0.62 ± 0.02 (mg/ml) β-carotene bleaching inhibition = 0.26 ± 0.03 (mg/ml)	GLSP have the most antioxidant activity when compared to the other polysaccharide extracts
		FB-Ps		DPPH scavenging activity = 0.22 ± 0.03 (mg/ml) Reducing power = 0.81 ± 0.03 (mg/ml) β-carotene bleaching inhibition = 9.03 ± 0.56 (mg/ml)	
		S-Ph		DPPH scavenging activity = 0.58 ± 0.04 (mg/ml) Reducing power = 1.25 ± 0.04 (mg/ml) β-carotene bleaching inhibition = 1.61 ± 0.21 (mg/ml)	
		S-ps		DPPH scavenging activity = 0.15 ± 0 (mg/ml) Reducing power = 0.69 ± 0.02 (mg/ml) β-carotene bleaching inhibition = 2.02 ± 0.29 (mg/ml)	
Nayak et al., (2021) [84]	Antimicrobial activity against P. intermedia	Mycelium		Minimum inhibitory concentration = 5.64 ± 8.5 (µg/ml)	The antimicrobial activity of mycelium and spore of G. lucidum was comparable
		Spore		Minimum inhibitory concentration = 3.62 ± 4.23 (µg/ml) (p = 0.9476. vs mycelium)	
Nayak et al., (2015) [85]	Antimicrobial activity	BSGWE	500 µg/ml	Percentage of sensitive = 65 (%) Percentage of resistant = 35 (%)	At 16-500 µg/ml *G. lucidum*, 65% of organisms were sensitive and 35% were resistant
			16 µg/ml	Percentage of sensitive = 65 (%) Percentage of resistant = 35 (%)	
Nayak et al., (2010) [83]	Antimicrobial activity	BSGWE		Minimum inhibitory concentration (*Staphylococcus aureus*) = 125 (µg/ml) Minimum inhibitory concentration (*Escherichia coli*) = 125 (µg/ml) Minimum inhibitory concentration (*Enterococcus faecalis*) < 2 (µg/ml) Minimum inhibitory concentration (*Klebsiella pneumoniae*) = 62.5 (µg/ml)	BSGWE displayed antibacterial activity
Shen et al., (2020) [18]	Antibacterial, antioxidant and anti-cancer	GLSP	600 µg/ml	DPPH radical-scavenging activities = 61.08 ± 1.22 (%) ^(*)^	The extracted triterpenoids have demonstrated the ability to inhibit DPPH radicals, antibacterial and anticancer
			800 µg/ml	(L929 cell) Cell viability = 82.68 ± 0.52 (%) ^(*)^ (HeLa cell) Cell viability = 51.77 ± 0.74 (%) ^(*)^	
			6 µl	The average inhibition zone diameter for *E. coli* = 11.04 ± 0.12 (mm) (p < 0.05 vs. control) ^(*)^ The average inhibition zone diameter for *S. aureus* = 11.74 ± 0.20 (mm) (p < 0.05 vs. control) ^(*)^	
			8 µl	The average inhibition zone diameter for *E. coli *= 11.69 ± 0.05 (mm) (p < 0.05 vs. control) ^(*)^ The average inhibition zone diameter for *S. aureus *= 11,83 ± 0.14 (mm) (p < 0.05 vs. control) ^(*)^	
			0	The average inhibition zone diameter for *E. coli =* 9.10 ± 0.11 (mm) ^(*)^ The average inhibition zone diameter for *S. aureus* = 9,13 ± 0.09 (mm) ^(*)^	
Zhu et al., (2018) [87]	Antimicrobial activity	GLSP		Inhibition zone diameter *E. coli* = 0 (mm) Inhibition zone diameter S. aureus = 0 (mm)	Chitosan obtained through both processes shows antibacterial potential
		C-T (surface chitosan obtained using thermochemical deacetylation)		Inhibition zone diameter *E. coli* = 16.9 ± 0.1 (mm) Inhibition zone diameter S. aureus = 16.4 ± 0.2 (mm)	
		C-U (surface chitosan obtained using ultrasound-assisted deacetylation)		Inhibition zone diameter *E. coli *= 23.8 ± 0.1 (mm) Inhibition zone diameter S. aureus = 21.3 ± 0.1 (mm)	
		C-C (commercial chitosan)		Inhibition zone diameter *E. coli *= 43.8 ± 0.2 (mm) Inhibition zone diameter S. aureus = 21.1 ± 0.3 (mm)	
Zhu et al., (2019) [31]	Hyperglycemic, antitumor and antioxidant activity	Proteoglycan-C	1 mg/ml	DPPH 90.6 ± 8.5 (%) ^(*)^ ABTS 73.3 ± 6.7 (%) ^(*)^	Proteoglycan-UC has stronger hypoglycemic and anti-bacterial effects
		Proteoglycan-UC	1 mg/ml	DPPH 72.6 ± 3.7 (%) ^(*)^ ABTS 47.2 ± 5.9 (%) ^(*)^	
		Control		Glucose concentration = 10.9 ± 0.78 (mmol/L) ^(*)^	
		Metformin	10^-3^ mol/l	Glucose concentration = 10.55 ± 0.87 (mmol/L) ^(*)^	
		Proteoglycan-C	10 mg/ml	Glucose concentration = 9.85 ± 0.66 (mmol/L) ^(*)^	
			1 mg/ml	Glucose concentration = 10.2 ± 0.52 (mmol/L)^ (*)^	
			0.1 mg/ml	Glucose concentration = 10.94 ± 0.48 (mmol/L) ^(*)^	
		Proteoglycan-UC	10 mg/ml	Glucose concentration = 9.98 ± 0.74 (mmol/L)^ (*)^	
			1 mg/ml	Glucose concentration = 10.42 ± 0.78 (mmol/L)^ (*)^	
			0.1 mg/ml	Glucose concentration = 10.98 ± 0.35 (mmol/L)^ (*)^	
		Proteoglycan-C		Inhibition zone diameter E. coli = 20.8 (mm) ^(*)^ Inhibition zone diameter S. aureus = 27.2 (mm) ^(*)^	
		Proteoglycan-UC		Inhibition zone diameter E. coli = 20.1 (mm) ^(*)^ Inhibition zone diameter S. aureus = 25.2 (mm) ^(*)^	
Yang et al., (2020) [92]	Prebiotic effects	Inulin		Growth rate at pH 2.5 in 0-2h = 0.086 (%) Growth rate at pH 2.5 in 2-4h = 0.043 (%)	Lactobacillus showed a better growth rate when using UB-O80 and B-O80 than with inulin
		UB-O80		Growth rate at pH 2.5 in 0-2h = 0.114 (%) Growth rate at pH 2.5 in 2-4h = 0.712 (%)	
		B-O80		Growth rate at pH 2.5 in 0-2h = 0.121 (%) Growth rate at pH 2.5 in 2-4h = 0.695 (%)	
Li et al., (2020) [79]	Induced intestinal barrier injury	SGPL + PTX (4 µM)	100 µg/ml	Apoptosis = 35.09 ± 2.9 (%)	SGP showed a potential protective effect against PTX-induced small intestine barrier damage
		SGPM + PTX (4 µM)	200 µg/ml	Apoptosis = 28.07 ± 5.37 (%)	
		SGPH + PTX (4 µM)	400 µg/ml	Apoptosis = 23.12 ± 1.66 (%) (p < 0.05 vs. PTX group)	
		PTX (4 µM)		Apoptosis = 35.90 ± 3.8 (%)	
Wang et al., (2012) [17]	Induced apoptosis in human leukemia THP-1 cells	GSP	0	Apotosis rate % = 2.06	LY294002 (Akt inhibitor) or PD98059 (ERK1/2 inhibitor) significantly enhanced active lipids of GLS-induced apoptosis in THP-1 cells
		GSP	1mg/ml	Apotosis rate % = 49.48 ± 4.88	
		GSP+DEVD		Apotosis rate % = 29.38 ± 2.06 (p < 0.01 compared with that of Ganoderma lucidum alone)	
		GSP+IETD		Apotosis rate % = 36.08 ± 4.13 (p < 0.05 compared with that of Ganoderma lucidum alone)	
		GSP+LEHD		Apotosis rate % = 25.77 ± 3.61 (p < 0.01 compared with that of Ganoderma lucidum alone)	
Wang et al., (2014) [82]	Inhibitive effect on apoptosis	Model	0 mg/mL	Apoptotic rate (TUNEL) (%) = 10.1 ± 0.55 (%)	In comparison to the moderate-dose, low-dose, and the model group, the apoptosis rate in the high dosage group was significantly lower
		Blank control group	0 mg/mL	Apoptotic rate (TUNEL) (%) = 1.84 ± 0.66 (%)	
		Drug control group	150 mg/mL	Apoptotic rate (TUNEL) (%) = 2.23 ± 0.82 (%)	
		High dose group	150 mg/mL	Apoptotic rate (TUNEL) (%) = 2.4 ± 0.61 (%)	
		Moderate dose group	100 mg/mL	Apoptotic rate (TUNEL) (%) = 4.63 ± 0.88 (%)	
		Low dose group	50 mg/mL	Apoptotic rate (TUNEL) (%) = 6.52 ± 1.02 (%)	
		Model	0 mg/mL	Splenic index (mg/g) = 2.6 ± 0.21	
		Blank control group	0 mg/mL	Splenic index (mg/g) = 3.87 ± 0.61	
		Drug control group	150 mg/mL	Splenic index (mg/g) = 3.92 ± 0.63	
		High dose group	150 mg/mL	Splenic index (mg/g) = 3.14 ± 0.36	
		Moderate dose group	100 mg/mL	Splenic index (mg/g) = 2.85 ± 0.34	
		Low dose group	50 mg/mL	Splenic index (mg/g) = 2.76 ± 0.63	
Pan et al., (2019) [81]	Protects bone marrow mesenchymal stem cells and hematopoiesis	DMSO	50 mg/mL	Apoptosis rate = 12.3 ± 1.6 (%) ^(*)^	GSL pre-treatment and co-treatment increased the proliferation and decreased the apoptosis in CTX-treated MSCs
		CTX		Apoptosis rate = 70.1 ± 15.17 (%) (p < 0.05 vs. DMSO) ^(*)^	
		Co-treated		Apoptosis rate = 35.04 ± 8.97 (%) (p < 0.05 vs. DMSO, p < 0.05 vs. CXT) ^(*)^	
		Pre-treated		Apoptosis rate = 25.23 ± 1.67 (%) (p < 0.05 vs. DMSO, p < 0.01 vs. CXT) ^(*)^	
		DMSO		CFU-E = 15.77 ± 2.2	
		CTX		CFU-E = 3.5 ± 0.54	
		Co-treated		CFU-E = 4.96 ± 0.57	
		Pre-treated		CFU-E = 11.33 ± 1.35	
		DMSO		BFU-E = 45.6 ± 2.58	
		CTX		BFU-E = 3.66 ± 0.98	
		Co-treated		BFU-E = 10.86 ± 1.17	
		Pre-treated		BFU-E = 35.9 ± 2.75	
		DMSO		CFU-GM = 91.06 ± 12.05	
		CTX		CFU-GM = 22.2 ± 3.65	
		Co-treated		CFU-GM = 31.43 ± 10.22	
		Pre-treated		CFU-GM = 52.1 ± 7.41	
Weng et al., (2010) [100]	Anti-aging	Untreated		Viability = 8.2 (%) ^(*)^	Ganodermasides A and B regulated UTH1 expression in order to extend the replicative life span of yeast
		Resveratrol	10 µM	Viability = 11 (%) ^(*)^	
		Ganodermaside A	1 µM	Viability = 8.9 (%) ^(*)^	
			10 µM	Viability = 11.4 (%) ^(*)^	
			100 µM	Viability = 9.4 (%) ^(*)^	
		Ganodermaside B	1 µM	Viability = 9.1 (%) ^(*)^	
			10 µM	Viability = 11.1 (%) ^(*)^	
			100 µM	Viability = 9.6 (%) ^(*)^	
Huang et al., (2011) [95]	Induced the activity of PPARα	DMSO		PPAR-α fold induction = 0.98 ± 0.26 ^(*)^	GLS induced the expression of PPAR-α target gene carnitine palmitoyl transferase-1a in human carcinoma HepG2 cells
		Wy14,643	50 μM	PPAR-α fold induction = 4.1 ± 0.15 (p < 0.001 vs. control) ^(*)^	
		GS	0.01 %	PPAR-α fold induction = 1.97 ± 0.21 (p < 0.01 vs. control) ^(*)^	
		GS	0.10 %	PPARα fold induction = 6.28 ± 0.36 (p < 0.001 vs. control) ^(*)^	
Li et al., (2013) [96]	Enhance of embryonic stem cells	GLS	0.01 %	% Change in Specific Growth Rate = 10.5% (p < 0.05)	GLS showed potential to improve mES cell proliferation
			0.10 %	% Change in Specific Growth Rate = 7.7% (p < 0.01)	
Wang et al., (2013) [97]	Anti-epileptic effects	Control		The expression level of NT-4 = 0.56 ± 0.31 ^(*)^	The expression of neurotrophin-4 was significantly increased in the GLS treated group compared with the model group
		Model		The expression level of NT-4 = 0.73 ± 0.28 ^(*)^	
		GLS group 1		The expression level of NT-4 = 1 ± 0.21 ^(*)^	
		GLS group 2		The expression level of NT-4 = 0.78 ± 0.35 ^(*)^	
Yang et al., (2016) [98]	Anti-epileptic effects	Normal control		Apoptosis rate = 8.6 ± 2.42	GAs could exert a protective effect on hippocampal neurons by promoting neuronal survival and the recovery of injured neurons
		Model group		Apoptosis rate = 54.4 ± 0.08 (p < 0.05 vs. normal control group)	
		L-GAs		Apoptosis rate = 25.65 ± 0.405 (p < 0.05 vs. model group)	
		M-GAs		Apoptosis rate = 19.85 ± 6.125 (p < 0.01 vs. other concentrations of GAs groups)	
		H-GAs		Apoptosis rate = 32.25 ± 0.845 (p < 0.01 vs. other concentrations of GAs groups)	
		Normal control		BDNF fluorescence intensity = 0.609 ± 0.073	
		Model group		BDNF fluorescence intensity = 0.679 ± 0.063 (P<0.05 vs normal control group)	
		L-GAs		BDNF fluorescence intensity = 0.756 ± 0.059 (P<0.05 vs model group)	
		M-GAs		BDNF fluorescence intensity = 0.916 ± 0.063 (P<0.01 vs other concentrations of GAs groups)	
		H-GAs		BDNF fluorescence intensity = 0.85 ± 0.065 (P<0.01 vs other concentrations of GAs groups)	
		Normal control		TRPC3 fluorescence intensity = 0.662 ± 0.05	
		Model group		TRPC3 fluorescence intensity = 0.767 ± 0.091 (P<0.05 vs normal control group)	
		L-GAs		TRPC3 fluorescence intensity = 0.85 ± 0.065 (P<0.05 vs model group)	
		M-GAs		TRPC3 fluorescence intensity = 0.925 ± 0.065 (P<0.01 vs other concentrations of GAs groups)	
		H-GAs		TRPC3 fluorescence intensity = 0.913 ± 0.088 (P<0.01 vs other concentrations of GAs groups)	
in vivo					
Chen et al., (2016) [41]	Antitumor effect in mice (n = 10)	Ganoderma extracts	4 g/kg	Inhibitory rate (S180 cells) = 39.1 (%) (p < 0.05 vs. control) Inhibitory rate (H22 cells) = 44.6 (%) (p < 0.01 vs. control)	The proliferation of the S180 and H22 transplant tumors in mice was significantly inhibited by Ganoderma spores
		Ganoderma spores oil	1.2 g/kg	Inhibitory rate (S180 cells) = 30.9 (%) (p < 0.05 vs. control) Inhibitory rate (H22 cells) = 44.9 (%) (p < 0.01 vs. control)	
		5-FU (positive control)	25 mg/kg	Inhibitory rate (S180 cells) = 54.1 (%) (p < 0.01 vs. control) Inhibitory rate (H22 cells) = 64.8 (%) (p < 0.01 vs. control)	
Chen et al., (2016) [36]	Antitumor effect in mice (n = 10)	E/E-SBGS	200 mg/kg daily	Tumor volume (A549 cells) = 831.35 ± 112.43 (mm^3^) (p < 0.05 vs. control)^ (*) (#)^ Tumor weight (A549 cells) = 0.9 ± 0.17 (g) (p < 0.05 vs. control)^ (*) (#)^	These results demonstrated that G. lucidum spores inhibited the growth of tumors
		Control		Tumor volume (A549 cells) = 1410.81 ± 216.22 (mm^3^)^ (*) (#)^ Tumor weight (A549 cells) = 1.54 ± 0.27 (g)^ (*) (#)^	
Dai et al., (2021) [44]	Antitumor effect in mice (n = 7)	40 nm-GLSO@NEs	3 ml/kg	Tumor weight (MGC803 cells) = 0.65 ± 0.31 (g) (p < 0.05 vs. control) ^(*)^	Tumors growth were significantly inhibited by 40 nm-GLSO@NEs
		Control		Tumor weight (MGC803 cells) = 1.63 ± 0.25 (g) ^(*)^	
Jiao et al., (2020) [42]	Antitumor effect in mice (n = 12)	Model		% apoptosis area = 4.89 ± 0.1 Fold change of control = 1 ± 0.1 Fold change of control = 1 ± 0.02	GLSO significantly inhibited the growth of 4T1 tumors in vivo
		Model (procaspase-9)		Fold change of control = 1 ± 0.1	
		GLSO (PPAR)	6g/kg/day	Fold change of control = 0.5 ± 0.2 (p < 0.05 vs. control)	
		GLSO	6g/kg/day	% apoptosis area = 17.4 ± 2.6 (p < 0.001 vs. model) Fold change of control = 0.7 ± 0.1 (p < 0.05 vs. control) Fold change of control = 0.9 ± 0.06	
		PTX		% apoptosis area = 11.24 ± 2.1 (p < 0.001 vs. model)	
Li et al., (2017) [35]	Antitumor effect in mice (n = 12)	Model		Tumor weight = 0.85 ± 0.01 (g) Liver weight = 1.24 (g)	In nude mice, consumption of 75 and 150 mg/kg BSGEE significantly lowered the growth of the HCT116 xenograft tumor
		Normal		Liver weight = 1.5 ± 1.17 (g)	
		BSGEE	75 mg/kg	Tumor weight = 0.59 ± 0.01 (g) (p < 0.05 vs. model) Liver weight = 1.24 (g)	
			150 mg/kg	Tumor weight = 0.37 ± 0.11 (g) (p < 0.01 vs. model) Liver weight = 1.46 (g)	
Na et al., (2017) [26]	Antitumor effect in mice (n = 18)	BSGWE	150 mg/kg	Tumor weight = 1.27 ± 0.19 (g) (p < 0.05 vs. control)	Final tumor weights of the two dosages were all significantly lower than those of the control group
			300 mg/kg	Tumor weight = 1.00 ± 0.21 (g) (p < 0.05 vs. control)	
		Control		Tumor weight = 2.22 ± 0.11 (g)	
		5-FU (n = 8)	20 mg/kg	Tumor weight = 1.28 ± 0.23 (g) (p < 0.05 vs. control)	
Shi et al., (2021) [39]	Antitumor effect in zebrafish (n = 30)	Cisplatin	50 µg/ml	Inhibition rate of human gastric cancer (SGC-7901) = 36.9 ± 3.12 (%) (p < 0.001 vs. model group) Inhibition rate of of human lung cancer (A549) = 31.91 ± 3.23 (%) (p < 0.001 vs. model group)	Compared to BSGP, RSGP displayed stronger inhibitory actions against tumors transplanted into zebrafish
		BGSP	33 µg/ml	Inhibition rate of human gastric cancer (SGC-7901) = 37.69 ± 4.37 (%) Inhibition rate of of human lung cancer (A549) = 13.47 ± 3.45 (%)	
			100 µg/ml	Inhibition rate of human gastric cancer (SGC-7901) = 50 ± 5.96 (%) (p < 0.01 vs. model group) Inhibition rate of of human lung cancer (A549) = 26.24 ± 3.26 (%) (p < 0.01 vs. model group)	
		RGSP	28 µg/ml	Inhibition rate of human gastric cancer (SGC-7901) = 50 ± 5.96 (%) (p < 0.01 vs. model group) Inhibition rate of of human lung cancer (A549) = 20 ± 5.16 (%)	
			83 µg/ml	Inhibition rate of human gastric cancer (SGC-7901) = 65.87 ± 3.57 (%) (p < 0.001 vs. model group) Inhibition rate of of human lung cancer (A549) = 26.8 ± 2.41 (%) (p < 0.01 vs. model group)	
			250 µg/ml	Inhibition rate of human gastric cancer (SGC-7901) = 76.98 ± 3.66 (%) (p < 0.001 vs. model group) Inhibition rate of of human lung cancer (A549) = 30.64 ± 1.84 (%) (p < 0.001 vs. model group)	
Su et al., (2018) [23]	Antitumor effect in mice (n = 6-8)	Model		Tumor = 522.19 ± 44.81 (mg) %T cell (CD3^+^) = 41.75 ± 2.04 (%) (p < 0.01 vs. norma lgroup) %Th cell (CD3^+^CD4^+^) = 28.7 ± 1.48 (%) %Tc cell (CD3^+^CD4^+^) = 8.81 ± 1.44 (%) Relative fold of change of pg1 protein = 0.5 ± 0.09 (%) (p < 0.01 vs. normal group) Relative fold of change of pg1 protein = 3.48 ± 0.7 (%) (p < 0.05 vs. model group) Chao1 index = 1257.73 ± 71.27 ACE index = 1283.42 ± 95.58	Polysaccharide-rich extract from BSG might be a good candidate for breast cancer treatment.
		PTX	15mg/mg	Tumor = 196.26 ± 44.74 (mg) (p < 0.01 vs. model group) %T cell (CD3^+^) = 26.86 ± 4.08 (%) (p < 0.01vs model group) %Th cell (CD3^+^CD4^+^) = 16.48 ± 3.89 (%) %Tc cell (CD3^+^CD4^+^) = 5.94 ± 1.01 (%) Relative fold of change of pg1 protein = 0.46 ± 0.08 (%) Relative fold of change of pg1 protein = 3.48 ± 0.7 (%) (p < 0.05 vs. model group)	
		ESGH	400mg/kg	Tumor = 371.49 ± 31.54 (mg) (p < 0.05 vs. model group) %T cell (CD3^+^) = 37.08 ± 3.67 (%) %Th cell (CD3^+^CD4^+^) = 22.03 ± 2.59 (%) %Tc cell (CD3^+^CD4^+^) = 11.11 ± 0.64 (%) Relative fold of change of pg1 protein = 0.54 ± 0.05 (%) Relative fold of change of pg1 protein = 0.63 ± 0.12 (%) (p < 0.01 vs. model group) Chao1 index = 1020.61 ± 143.39 (p < 0.01 vs. normal group) ACE index = 1101.6 ± 106.4 (p < 0.01 vs. normal group)	
		ESGL	200mg/kg	Tumor = 445.09 ± 49.06 (mg) %T cell (CD3^+^) = 37.96 ± 2.62 (%) %Th cell (CD3^+^CD4^+^) = 24.62 ± 1.86 (%) %Tc cell (CD3^+^CD4^+^) = 13.18 ± 1.58 (%) Eelative fold of change of pg1 protein = 0.51 ± 0.03 (%) Eelative fold of change of pg1 protein = 0.78 ± 0.09 (%) (p < 0.01 vs. model group)	
		Normal		%T cell (CD3^+^) = 62.18 ± 2.63 (%) %Th cell (CD3^+^CD4^+^) = 44.62 ± 2.38 (%) %Tc cell CD3^+^CD4^+^) = 15.05 ± 1.07 (%) Relative fold of change of pg1 protein = 1.16 ± 0.09 (%) Relative fold of change of pg1 protein = 1.21 ± 0.18 (%) Chao1 index = 1391.75 ± 123.25 ACE index = 1497.32 ± 116.68	
Su et al., (2018) [28]	Antitumor effect in mice (n = 6)	Model		Tumor = 0.81 ± 0.24 T cell (CD3+) = 52.5 ± 7.5 (%) PD-1 T cell = 21.25 ± 5.75 (%) Tim-3 T cell = 16.6 ± 6.7 (%) Tc cell CD3+CD8+ = 25.56 ± 5.74 (%) (p < 0.01) Th cell CD3+CD4+ = 12.62 ± 1.38 (%) Chao1 index = 2323.8 ± 380.2 ACE index = 2457.14 ± 322.86	The combination of PTX and SGP demonstrated superior tumor control in the mouse breast cancer model, with early tumor growth reduction and clear ki67 expression inhibition than PTX alone
		PTX		Tumor = 0.64 ± 0.15 (p < 0.05 vs. model group) T cell (CD3+) = 55 ± 8.3 (%) PD-1 T cell = 20.83 ± 6.25 (%) Tim-3 T cell = 22.5 ± 9.1 (%) (p < 0.05) Tc cell CD3+CD8+ = 27.6 ± 7 (%) (p < 0.01) Th cell CD3+CD4+ = 10.67 ± 1.95 (%) (p < 0.05) Chao1 index = 1885.71 ± 380.29 (p < 0.05) ACE index = 1866.6 ± 380.4 (p < 0.05)	
		SLP		Tumor = 0.52 ± 0.12 (p < 0.05 vs. model group) T cell (CD3+) = 47.5 ± 9.1 (%) PD-1 T cell = 14.9 ± 5.1 (%) Tim-3 T cell = 14.9 ± 6.7 (%) (p < 0.01) Tc cell CD3+CD8+ = 21.03 ± 7.01 (%) (p < 0.01) Th cell CD3+CD4+ = 9.9 ± 2.13 (%) Chao1 index = 1809.52 ± 190.48 (p < 0.05) ACE index = 1733.3 ± 361.7 (p < 0.05)	
		SHP		Tumor = 0.44 ± 0.2 (p < 0.05 vs. model group) T cell (CD3+) = 47.5 ± 8.33 (%) PD-1 T cell = 14.16 ± 5 (%) Tim-3 T cell = 13.3 ± 4.2 (%) Tc cell CD3+CD8+ = 18.14 ± 6.18 (%) Th cell CD3+CD4+ = 10.29 ± 1.94 (%) ACE index = 1504.76 ± 228.24 (p < 0.05)	
Zhang et al., (2019) [25]	Antitumor effect in mice	NC		Tumor volume = 2.21 ± 0.28 (mm^3^) Tumor weight = 1.86 ± 0.07 (g)	BSGWE significantly inhibited tumor growth
		BSGWE	600 mg/kg	Tumor volume = 1.14 ± 0.67 (mm^3^) (p < 0.01 vs. control) Tumor weight = 1.61 ± 0.14 (g) p < 0.01 vs. control)	
Pan et al., (2019) [27]	Antitumor effect in mice (n =10)	Control		Tumor weight = 3 ± 0.4 (g) Tumor volume 6 weeks = 1722.97 ± 185.81 (mm^3^)	GLP inhibited tumor growth
		GLP	150 mg/kg	Tumor weight = 1.92 ± 0.3 (g) Tumor volume 6 weeks = 1283.78 ± 168.92 (mm^3^)	
			300 mg/kg	Tumor weight = 1.25 ± 0.2 (g) Tumor volume 6 weeks = 979.72 ± 168.92 (mm^3^)	
Wang et al., (2012) [29]	Antitumor effect in mice (n =10)	Model		Inhibitory ratio (Sarcoma 180 cells) = 0 (%)	BSGP 100 and 200 mg/kg significantly decreased the growth of sarcoma 180 in comparison to the model group
		BSGP	50 mg/kg	Inhibitory ratio (Sarcoma 180 cells) = 30.7 (%)	
			100mg/kg	Inhibitory ratio (Sarcoma 180 cells) = 49.1 (%)	
			200mg/kg	Inhibitory ratio (Sarcoma 180 cells) = 59.9 (%)	
		CY	30mg/kg	Inhibitory ratio (Sarcoma 180 cells) = 81 (%)	
He et al., (2020) [24]	Antitumor effect in mice (n = 3)	NC	200 μL saline	Tumor volume (1st week) = 0.31 (mm^3^) Tumor volume (2nd week) = 0.71 (mm^3^) Tumor volume (3rd week) = 1.64 (mm^3^) Tumor volume (4th week) = 3.14 (mm^3^)	BSGWE inhibited tumor growth
		BSGWE	0.5 mg BSGWE dissolved in 100 μL saline	Tumor volume (1st week) = 0.31 (mm^3^) (p < 0.001 vs. control) Tumor volume (2nd week) = 0.57 (mm^3^) (p < 0.001 vs. control) Tumor volume (3rd week) = 1.37 (mm3) (p < 0.001 vs. control) Tumor volume (4th week) = 2.49 (mm^3^) (p < 0.001 vs. control)	
Guo et al., (2009) [54]	Antitumor effect in C57BL/6 and BALB/c nu/nu mice (n = 10)	GSG	50 mg/kg	(C57BL/6 mice) Tumor weight = 702.61 ± 60 (mg) (p < 0.05 vs. negative control) ^(*)^ (BALB/c nu/nu) Tumor weight = 976.63 ± 67 (mg) ^(*)^	GSG administration increased the anti-tumor activity that had been identified against lung carcinoma in Lewis mice
			100 mg/kg	(C57BL/6 mice) Tumor weight = 562 ± 41 (mg) (p < 0.05 vs. negative control) ^(*)^ (BALB/c nu/nu) Tumor weight = 969.5 ± 55 (mg) ^(*)^	
			200 mg/kg	(C57BL/6 mice) Tumor weight = 412 ± 44 (mg) (p < 0.05 vs. negative control) ^(*)^ (BALB/c nu/nu) Tumor weight = 969.5 ± 55 (mg) ^(*)^	
		Cyclophosphamide		(C57BL/6 mice) Tumor weight = 19 ± 22 (mg) (p < 0.01 vs. negative control) ^(*)^ (BALB/c nu/nu) Tumor weight = 52.27 ± 21 (mg) (p < 0.01 vs. negative control) ^(*)^	
		PBS (NC)		(C57BL/6 mice) Tumor weight = 891 ± 62 (mg) ^(*)^ (BALB/c nu/nu) Tumor weight = 973.63 ± 64 (mg) ^(*)^	
Yue et al., (2008) [38]	Antitumor effect in mice (n = 19)	Control		Tumor weight = 426.1 ± 172 (mg)	2 and 4 g/kg of BS were significantly different from those of the untreated control mice
		BS	1000 mg/kg	Tumor weight = 330.5 ± 191.4 (mg) (p < 0.05 vs. control)	
		BS	2000 mg/kg	Tumor weight = 305 ± 184 (mg) (p < 0.05 vs. control)	
		BS	4000 mg/kg	Tumor weight = 329.9 ± 195.8 (mg)	
Fu et al., (2019) [34]	Antitumor effect in mice (n = 8)	Control	0.1 mL/10g BW	Tumor weight = 1.45 ± 0.24 (g)	WGLP could significantly inhibit the S180 tumor growth
		CTX	30mg/kg BW	Tumor weight = 0.88 ± 0.4 (g) (p < 0.01 vs. control)	
		WGLP	3mg/kg BW	Tumor weight = 0.96 ± 0.29 (g) (p < 0.05 vs. control)	
			10mg/kg BW	Tumor weight = 0.84 ± 0.32 (g) (p < 0.01 vs. control)	
			30mg/kg BW	Tumor weight = 0.82 ± 0.34 (g) (p < 0.01 vs. control)	
			100 mg/kg BW	Tumor weight = 0.86 ± 0.16 (g) (p < 0.01 vs. control)	
Liu et al., (2002) [22]	Antitumor effect in mice (n = 10)	Normal saline (negative control)	20 ml/kg per day	Tumor weight hepatoma cell = 2.17 ± 0.16 (g) Tumor weight sarcoma S-180 cell = 1.78 ± 0.13 (g) Tumor weight sarcoma L-II cell = 2.21 ± 0.21 (g)	Both the oil extract from the germinating spores and the SBGS had notable anticancer effects
		CTX (positive control)	20 ml/kg per day	Tumor weight hepatoma cell = 0.8 ± 0.14 (g) (p < 0.001 vs. negative control) ^(*)^ Tumor weight sarcoma S-180 cell = 0.37 ± 0.1 (g) (p < 0.001 vs. negative control) ^(*)^ Tumor weight sarcoma L-II cell = 0.68 ± 0.18 (g) (p < 0.001 vs. negative control) ^(*)^	
		Spore	8 g/kg per day in twice	Tumor weight hepatoma cell = 1.79 ± 0.28 (g) (p < 0.001 vs. negative control) ^(*)^ Tumor weight sarcoma S-180 cell = 1.44 ± 0.22 (g) (p < 0.001 vs. negative control) ^(*)^ Tumor weight sarcoma L-II cell = 1.83 ± 0.29 (g) (p < 0.001 vs. negative control) ^(*)^	
		GS	8 g/kg per day in twice	Tumor weight hepatoma cell = 1.39 ± 0.27 (g) (p < 0.001 vs. negative control) ^(*)^ Tumor weight sarcoma S-180 cell = 1.13 ± 0.22 (g) (p < 0.001 vs. negative control) ^(*)^ Tumor weight sarcoma L-II cell = 1.42 ± 0.26 (p < 0.001 vs. negative control) ^(*)^	
		SBGS	2 g/kg per day	Tumor weight hepatoma cell = 1.18 ± 0.17 (g) (p < 0.001 vs. negative control) ^(*)^ Tumor weight sarcoma S-180 cell = 0.8 ± 0.17 (g) (p < 0.001 vs. negative control) ^(*)^ Tumor weight sarcoma L-II cell = 0.98 ± 0.2 (p < 0.001 vs. negative control) ^(*)^	
		SBGS	4 g/kg per day	Tumor weight hepatoma cell = 0.92 ± 0.13 (g) (p < 0.001 vs. negative control) ^(*)^ Tumor weight sarcoma S-180 cell = 0.45 ± 0.15 (g) (p < 0.001 vs. negative control) ^(*)^ Tumor weight sarcoma L-II cell = 0.67 ± 0.13 (p < 0.001 vs. negative control) ^(*)^	
		SBGS	8 g/kg per day in twice	Tumor weight hepatoma cell = 0.39 ± 0.13 (g) (p < 0.001 vs. negative control) ^(*)^ Tumor weight sarcoma S-180 cell = 0.25 ± 0.09 (g) (p < 0.001 vs. negative control) ^(*)^ Tumor weight sarcoma L-II cell = 0.37 ± 0.12 (p < 0.001 vs. negative control) ^(*)^	
		lipids	5 g/kg per day	Tumor weight hepatoma cell = 0.22 ± 0.1 (g) (p < 0.001 vs. negative control) ^(*)^ Tumor weight sarcoma S-180 cell = 0.15 ± 0.11 (g) (p < 0.001 vs. negative control) ^(*)^ Tumor weight sarcoma L-II cell = 0.23 ± 0.1 (p < 0.001 vs. negative control) ^(*)^	
Bao et al., (2002) [48]	Immunological activity in mice	PSGL-I-1A	25 mg/kg	A570 = 0.81 ± 0.13 (p < 0.01 vs. control) ^(*)^	The polysaccharide PSGL-I-1A showed a significantly enhancing effect on Concanavalin A-induced T lymphocyte proliferation
			50 mg/kg	A570 = 0.95 ± 0.15 (p < 0.001 vs. control) ^(*)^	
		CHC-1 (PC)	25 mg/kg	A570 = 0.7 ± 0.08 (p < 0.05 vs. control) ^(*)^	
			50 mg/kg	A570 = 0.78 ± 0.12 (p < 0.01 vs. control) ^(*)^	
		Negative control	0	A570 = 0.56 ^(*)^	
Bao et al., (2001) [49]	Immunological activity in mice (n =7)	PSG-CM-1	25 mg/kg	A570 (T cell) = 0.99 ± 0.01 (p < 0.001 vs. control) A570 (B cell) = 0.99 ± 0.02 (p < 0.001 vs. control)	Low degree of substitution carboxymethylated (1-3)-β-D-glucan significantly increased T and B lymphocyte proliferation, antibody production, and spleen tissue mass
			50 mg/kg	A570 (T cell) = 0.87 ± 0.01 (p < 0.01 vs. control) A570 (B cell) = 1.01 ± 0.01 (p < 0.001 vs. control)	
		PSG-CM-2	25 mg/kg	A570 (T cell) = 0.97 ± 0.03 (p < 0.01 vs. control) A570 (B cell) = 0.83 ± 0.01	
			50 mg/kg	A570 (T cell) = 0.97 ± 0.01 (p < 0.01 vs. control) A570 (B cell) = 0.88 ± 0.03 (p < 0.05 vs. control)	
		PSG-CM-3	25 mg/kg	A570 (T cell) = 0.71 ± 0.02 A570 (B cell) = 0.8 ± 0.04	
			50 mg/kg	A570 (T cell) = 0.84 ± 0.01 (p < 0.05 vs. control) A570 (B cell) = 0.82 ± 0.01	
		Negative control	0	A570 (T cell) = 0.68 ± 0.01 A570 (B cell) = 0.82 ± 0.01	
Bao et al., (2001) [56]	Immunological activity in mice (n =8) Immunological activity in mice (n =8)	PGL	25 mg/kg	A520 = 0.21 ± 0.14 (p < 0.001 vs. control) ^(*)^ A570 (T cell) = 0.81 ± 0.16 (p < 0.001 vs. control)^ (*)^ A570 (B cell) = 0.79 ± 0.11 (p < 0.05 vs. control)^ (*)^	The polysaccharide might significantly lower Concanavalin A or LPS-induced lymphocyte proliferation and antibody production
			50 mg/kg	A520 = 0.2 ± 0.14 (p < 0.001 vs. control)^ (*)^ A570 (T cell) = 0.59 ± 0.16 (p < 0.001 vs. control)^ (*)^ A570 (B cell) = 0.56 ± 0.11 (p < 0.01 vs. control)^ (*)^	
		Negative control	0	A520 = 0.38 ± 0.07 ^(*)^ A570 (T cell) = 1.09 ± 0.08 ^(*)^ A570 (B cell) = 0.89 ± 0.07 ^(*)^	
Bao et al., (2001) [57]	Immunological activity in mice (n =7)	SP		A520 = 1.23 ± 0.06 A570 (T cell) = 0.84 ± 0.06 (p < 0.05 vs. control) A570 (B cell) = 0.93 ± 0.02 (p < 0.01 vs. control) IgG = 18.9 ± 2 C-3 = 2.42 ± 0.12 (p < 0.05 vs. control)	The degraded glucan had immunological activities in view of the lymphocyte proliferation (T and B cells) and the production of antibodies against sheep red blood cells (SRBC) in mice
		SP-1		A520 = 1.21 ± 0.02 A570 (T cell) = 0.95 ± 0.02 (p < 0.001 vs. control) A570 (B cell) = 0.94 ± 0.01 (p < 0.01 vs. control) IgG = 19.7 ± 2.3 C-3 = 2.1 ± 0.36	
		Control		A520 = 1.11 ± 0.02 A570 (T cell) = 0.55 ± 0.02 A570 (B cell) = 0.6 ± 0.04 IgG = 17.3 ± 1.5 C-3 = 2.08 ± 0.35	
Li et al., (2020) [61]	Immunological activity in zebrafish (n = 10)	BGLS	22 (mcg/mL)	The number of neutrophils = 107.24 ± 3.76 (p < 0.05 vs. model) ^(*)^ Neutrophil recovery rate = 42.13 ± 5.95 (%) ^(*)^ The number of macrophage that phagocytized ACNP = 9.91 ± 1.2 ^(*)^ Macrophage formation efficiency = 0.67 ± 3.22 (%) ^(*)^ Macrophage phagocytosis efficiency = 17.8 ± 5.58 (%) ^(*)^	The triterpenes from *G. lucidum* increased immunomodulation and induced cell death to suppress lung cancer growth
		RGLS	33 (mcg/mL)	The number of neutrophils = 117.05 ± 8.06 (p < 0.01 vs. model) ^(*)^ Neutrophil recovery rate = 54.04 ± 11.91 (%) ^(*)^ The number of macrophage that phagocytized ACNP = 11.4 ± 0.53 (p < 0.01 vs. model) ^(*)^ Macrophage formation efficiency = 34.74 ± 6.61 (%) (p < 0.01) ^(*)^ Macrophage phagocytosis efficiency = 36.1 ± 3.05 (%) (p < 0.01) ^(*)^	
			1000 (mcg/mL)	The number of macrophage that phagocytized ACNP = 12.29 ± 0.5 (p < 0.001 vs. model) ^(*)^ Macrophage formation efficiency = 29.66 ± 4.07 (%) (p < 0.01) ^(*)^ Macrophage phagocytosis efficiency = 44.23 ± 4.58 (%) (p < 0.001) ^(*)^	
		Control		The number of neutrophils = 135.63 ± 4.12 ^(*)^	
		Model		The number of neutrophils = 73.59 ± 3.41 ^(*)^ The number of macrophage that phagocytized ACNP = 8.34 ± 0.3 ^(*)^	
Liu et al., (2021) [59]	Immunological activity in mice (n =10)	Control (water)	5 mg/kg per day	HC_50_ = 240.6 ± 11.8	GLSB50 and GLSB70 showed a significant increase in the HC50 value as well as the positive lentinan group
		Model (CTX)	5 mg/kg per day	HC_50_ = 155.54 ± 4.9 (p < 0.001 vs. control) ^(*)^	
		GLSB50	300 mg/kg per day	HC_50_ = 207.45 ± 5.9 (p < 0.01 vs. control; p < 0.05 vs. model) ^(*)^	
		GLSB70	300 mg/kg per day	HC_50_ = 200 ± 5.9 (p < 0.05 vs control ; p < 0.01 vs. model) ^(*)^	
		Lentinan	300 mg/kg per day	HC_50_ = 207.92 ± 10.9 (p < 0.01 vs control ; p < 0.05 vs. model) ^(*)^	
Su et al., (2021) [58]	Immunological activity in mice (n =8-10)	Normal		Thymus coeficiency = 0.12 ± 0.01 NK cell’s tumor-killing ability = 47.76 ± 2.24	Both CGLP and RPGS inhibited spleenocyte proliferative activity in response to mitogen, however only CGLP enhanced NK cell tumor-killing capacity
		LNT		Thymus coeficiency = 0.12 ± 0.007 NK cell’s tumor-killing ability = 40.29 ± 3.73	
		CGLP		Thymus coeficiency = 0.11 ± 0.002 (p < 0.05) (p < 0.05) NK cell’s tumor-killing ability = 76.86 ± 7.44 (p < 0.01)	
		RPGS		Thymus coeficiency = 0.11 ± 0.015 NK cell’s tumor-killing ability = 46.26 ± 2.99	
Wang et al., (2017) [62]	Immunological activity in mice (n =10)	Control group		Ear swelling = 6.6 ± 1.5 (mg) Weight of the right ear = 14.7 ± 1.4 (mg) Weight of the left ear = 8.1 ± 0.7 (mg)	GLSWA-I (300 mg/kg) administration reversed the decreasing of ear swelling of model group
		Model group		Ear swelling = 2.9 ± 1.2 (mg) (p < 0.01 vs. control group) Weight of the right ear = 10.7 ± 1.4 (mg) (p < 0.01 vs. control group) Weight of the left ear = 7.7 ± 0.6 (mg)	
		Lentinan	150 mg/kg	Ear swelling = 4.4 ± 0.8 (mg) (p < 0.05 vs. control group) Weight of the right ear = 11.7 ± 1.6 (mg) Weight of the left ear = 7.6 ± 1.1 (mg)	
		Low-dose GLSWA-I	75 mg/kg	Ear swelling = 4.2 ± 1.6 (mg) Weight of the right ear = 12.1 ± 1.6 (mg) Weight of the left ear = 7.9 ± 0.9 (mg)	
		Medium-dose GLSWA-I	150 mg/kg	Ear swelling = 4.6 ± 2.1 (mg) (p < 0.05 vs. control group) Weight of the right ear = 12.5 ± 2.4 (mg) Weight of the left ear = 7.8 ± 0.8 (mg)	
		High-dose GLSWA-I	300 mg/kg	Ear swelling = 4.8 ± 1.7 (mg) (p< 0.05 levels compared with the model group) Weight of the right ear = 12.4 ± 1.8 (mg) (p< 0.05 levels compared with the model group) Weight of the left ear = 7.6 ± 0.8 (mg)	
Wu et al., (2020) [60]	Immunological activity in mice (n = 6)	Control		Serum henolysin level = 490.44 ± 18.38 (HC_50_) ^(*)^ NK activity = 0.76 ± 0.07 (p < 0.05 vs. control) ^(*)^ Phagocytic index = 4.48 ± 0.25 (p < 0.05 vs. Control) ^(*)^ HC_50_ = 477.78 ± 22.22 ^(*)^	GLSO (at 800 mg/kg) improved the phagocytosis of macrophages and the cytotoxicity of NK cells in mice.
		GLSO_H	800 mg/kg	Serum henolysin level = 468.38 ± 84.56 (HC_50_) ^(*)^ NK activity = 1.05 ± 0.17 (p < 0.05 vs. control) ^(*)^ Phagocytic index = 4.88 ± 0.13 (p <0.05 vs. control) ^(*)^ HC_50_ = 455.56 ± 83.33 ^(*)^	
		GLSO_L	400 mg/kg	Serum henolysin level = 442.65 ± 91.91 (HC_50_) ^(*)^ NK activity = 0.93 ± 0.24 (p < 0.05 vs. control) ^(*)^ Phagocytic index = 4.75 ± 0.13 (p < 0.05 vs. control) ^(*)^ HC_50_ = 433.33 ± 83.33 ^(*)^	
Ma et al., (2009) [63]	Immunological activity in mice (n = 12)	Control	0.9% NaCl	Thymus weight = 141 ± 19 Con-A induced lymphocyte proliferation = 0.44 ± 0.14	Thymus weight of mice treated with BSGP and Cy combined was significantly higher than with Cy alone
		Cy	20 mg/kg/day	Thymus weight = 52 ± 24 (p < 0.01 vs. control) Con-A induced lymphocyte proliferation = 0.13 ± 0.07 (p < 0.01 vs. control)	
		GL-SP	50 mg/kg/day	Thymus weight = 117 ± 18 Con-A induced lymphocyte proliferation = 0.45 ± 0.14	
		Cy+GL-SP	20 mg/kg/day+50 mg/kg/day	Thymus weight = 75 ± 37 (p < 0.05 vs. control; p < 0.05 vs. Cy-treated group) Con-A induced lymphocyte proliferation = 0.18 ± 0.09 (p < 0.01 vs. control; p < 0.05 vs. Cy-treated group)	
Sang et al., (2021) [66]	Anti-inflammatory, anti-obesity (n = 6)	HFD-fed donors (control)		Body weight gain = 6.9 ± 0.97 (g)	BSGP reduced the obesity, hyperlipidemia, inflammation, and fat accumulation that caused by HFD in C57BL/6 J mice
		HFD BSGP	300 mg/kg	Body weight gain = 4.77 ± 0.36 (g) (p < 0.05 vs. control)	
		Control		TC (mmol/L) = 6 ± 0.23 LDL (mmol/L) = 1.18 ± 0.22 TNF-α (ng/L) = 1714.28 ± 95.23 IL-1β (ng/L) = 135.71 ± 4.76	
		BSGP	100 mg/kg	TC (mmol/L) = 5.36 ± 0.27 (p < 0.05 vs. control) LDL (mmol/L) = 0.7 ± 0.05 (p < 0.01 vs. control) TNF-α (ng/L) = 1190.48 ± 47.62 (p < 0.001 vs. control) IL-1β (ng/L) = 95.23 ± 9.52 (p < 0.001 vs. control)	
		BSGP	300 mg/kg	TC (mmol/L) = 5.72 ± 0.18 LDL (mmol/L) = 0.67 ± 0.03 (p < 0.01 vs. control) TNF-α (ng/L) = 1333.3 ± 47.62 (p < 0.01 vs. control) IL-1β (ng/L) = 78.57 ± 9.52 (p < 0.001 vs. control)	
Levin et al., (2017) [72]	Protection of bladder function following oxidative stress	Control		Bladder weight = 1,8 ± 0,2 (mg) Compliance = 0,5 ± 0,05 (cm H20/ 20% capacity)	These findings show that GLS provided superior bladder function protection following I/R (oxidative stress)
		Control GL		Bladder weight = 1,6 ± 0,2 (mg) Compliance = 0,4 ± 0,05 (cm H20/20% capacity) (significantly different from control, significantly different from control + I/R; p < 0.05)	
		I/R		Bladder weight = 2,4 ± 0,2 (mg) (p < 0.05 of control) Compliance = 4,5 ± 0,5 (cm H20/ 20% capacity) (significantly different from control)	
		I/R + GL		Bladder weight = 2,3 ± 0,2 (mg) Compliance = 1,2 ± 0,3 (cm H20/ 20% capacity) (significantly different from control + I/R; p < 0.05)	
Zhang et al., (2021) [73]	Antioxidant activity	Control		Mean life span (female) = 50.1 ± 0.55 (d) Maximum life span (female) = 61.93 ± 0.19 (d) Maximum life span (male) = 60.41 ± 0.2 (d) Mean life span (male) = 48.93 ± 0.44 (d) Mean life span (female) = 21.46 ± 0.58 (h) Maximum life span (female) = 32.2 ± 0.69 (h) Mean life span (male) = 21.14 ± 0.63 (h) Maximum life span (male) = 32.3 ± 0.92 (h)	GLSO increases the average lifespan of *Drosophila melanogaster*
		GLSO	0.3125 mg/ml	Mean life span (female) = 50.85 ± 0.53 (d) Maximum life span (female) = 63.87 ± 0.2 (d) (p < 0.001 vs. control) Mean life span (male) = 50.45 ± 0.52 (d) (p < 0.05 vs. control) Maximum life span (male) = 61.53 ± 0.17 (d) (p < 0.01 vs. control) Mean life span (female) = 22 ± 0.53 (h) Maximum life span (female) = 33.8 ± 0.69 (h) Mean life span (male) = 21.8 ± 0.61 (h) Maximum life span (male) = 34 ± 1.07 (h)	
			0.625 mg/ml	Mean life span (female) = 53.01 ± 0.49 (d) (p < 0.01 vs. control) Maximum life span (female) = 63.87 ± 0.2 (d) (p < 0.001 vs. control) Mean life span (male) = 52.01 ± 0.59 (d) (p < 0.001 vs. control) Maximum life span (male) = 62.53 ± 0.27 (d) (p < 0.001 vs. control) Mean life span (female) = 22.82 ± 0.6 (h) (p < 0.05 vs. control) Maximum life span (female) = 33.6 ± 1.02 (h) Mean life span (male) = 22.42 ± 0.64 (h) Maximum life span (male) = 34.2 ± 1.34 (h)	
			1.25 mg/ml	Mean life span (female) = 56.04 ± 0.64 (d) (p < 0.001 vs. control) Maximum life span (female) = 65.93 ± 0.23 (d) (p < 0.001 vs. control) Mean life span (male) = 53.89 ± 0.55 (d) (p < 0.001 vs. control) Maximum life span (male) = 63.62 ± 0.2 (d) (p < 0.001 vs. control) Mean life span (female) = 23.56 ± 0.63 (h) (p < 0.05 vs. control) Maximum life span (female) = 35.8 ± 0.95 (h) (p < 0.05 vs. control) Mean life span (male) = 23.8 ± 0.66 (h) (p < 0.05 vs. control) Maximum life span (male) = 37 ± 0.98 (h) (p < 0.01 vs. control)	
Zhan et al., (2016) [87]	Antimicrobial activity (n = 3)	Control		LogCFU week 5 (lung) = 0.6 ± 0.42 LogCFU week 5 (spleen) = 3.73 ± 0.14	A little amount of host defense against bacterial proliferation may be provided by G. lucidum extract when used before M. tuberculosis infection
		G. lucidum extract (therapy)	15 mg of GLS and 15 mg spore lipids	LogCFU week 5 (lung) = 1.38 ± 0.64 (p < 0.05 vs. control) LogCFU week 5 (spleen) = 3.54 ± 0.09 (p < 0.01 vs. control)	
Jiang et al., (2021) [88]	Glucose/lipid metabolism and gut microbiota in mice (n = 8)	NC		Blood glucose concentration (4W) = 6.2 ± 0.5 TG = 0.285 ± 0.0 HDL-C = 2.79 ± 0.1	EGLS significantly enhanced glycometabolism and lipometabolism parameters in type 2 diabetic mellitus rats
		MC		Blood glucose concentration (4W) = 32.2 ± 1.7 (p < 0.05) TG = 2.915 ± 1.2 (p < 0.05 vs. control) HDL-C = 2.79 ± 0.1 (p < 0.05 vs. control)	
		EGLS	10.5 g/kgbw/day	Blood glucose concentration (4W) = 24.6 ± 2.8 (p < 0.05) TG = 0.644 ± 1.7 (p < 0.05 vs. model) HDL-C = 2.79 ± 0.1 (p < 0.05 vs. model)	
Lai et al., (2020) [91]	Lipid-lowering and anti-atherosclerotic effects in rabbit (n = 9)	Control		TC/HDL-C ratio (week 4) = 2.5 ± 0.33 Hepatocyte steatosis (score) = 0 ± 0 (p < 0.05 vs. model)	EEG has lipid-lowering and anti-atherosclerotic effects through increasing the expression of genes related to reverse cholesterol transport and metabolism, including LXRa and downstream genes
		Model		TC/HDL-C ratio (week 4) = 5.13 ± 0.7 Hepatocyte steatosis (score) = 3.6 ± 0.5	
		EEG-L		TC/HDL-C ratio (week 4) = 5.14 ± 0.7 (p < 0.05 vs. model) Hepatocyte steatosis (score) = 3.7 ± 0.5	
		EEG-M		TC/HDL-C ratio (week 4) = 4.3 ± 0.86 (p < 0.05 vs. model) Hepatocyte steatosis (score) = 2.5 ± 0.5 (p < 0.05 vs. model)	
		EEG-H		TC/HDL-C ratio (week 4) = 3.63 ± 0.88 (p < 0.05 vs. model) Hepatocyte steatosis (score) = 0.8 ± 0.6 (p < 0.05 vs. model)	
		Atorvastatin		TC/HDL-C ratio (week 4) = 6.69 ± 1.47	
Shaher et al., (2020) [89]	Hyperglycemia-mediated cardiomyopathy protection in mice (n = 8)	Control	5 mL/kg saline	Body weight = 416 ± 22.46 (g) Blood glucose = 6.91 ± 0.34 HbA1C = 1.7 ± 0.13	When compared to the diabetic group without treatment, GLS significantly lowered glucose levels
		STZ	50 mg/kg streptozotocin	Body weight = 308 ± 12.81 (g) (p < 0.01 vs. control) Blood glucose = 30.08 ± 1.34 (p < 0.01 vs. control) HbA1C = 2.16 ± 0.21 (p < 0.01 vs. control)	
		STZ + GLS	50 mg/kg streptozotocin (i.p.) and 300 mg/kg GLS (p.o.)	Body weight = 334 ± 27.4 (g) (p < 0.01 vs. control) Blood glucose = 23.98 ± 1.28 (p < 0.01 vs. STZ) HbA1C = 2.03 ± 0.19 (p < 0.05)	
Wang et al., (2015) [90]	Glucose and lipid metabolisms in mice (n = 8)	Normal (control)		Blood glucose level 4 weeks = 6.2 ± 0.52 (mmol/L) TG = 0.29 ± 0 (mmol/L) TC = 2.92 ± 0.07 (mmol/L) HDL-C = 2.90 ± 0.07 (mmol/L)	When compared to the model control group, the diabetic rats in the GLSP group's level of lipids decreased significantly after 4 weeks
		Model		Blood glucose level 4 weeks = 32.22 ± 1.71 (mmol/L) (p < 0.05 vs. control) TG = 2.96 ± 0.27 (mmol/L) (p < 0.05 vs. control) TC = 5.57 ± 0.47 (mmol/L) (p < 0.05 vs. control) HDL-C = 1.32 ± 0.45 (mmol/L) (p < 0.05 vs. control)	
		GLSP		Blood glucose level 4 weeks = 24.31 ± 1.17 (mmol/L) (p < 0.05 vs. model) TG = 1.49 ± 0.55 (mmol/L) (p < 0.05 vs. model) TC = 4.58 ± 0.09 (mmol/L) (p < 0.05 vs. model) HDL-C = 2.57 ± 0.29 (mmol/L) (p < 0.05 vs. model)	
Gao et al., (2010) [74]	Inhibiting N-methyl-N-nitrosourea-induced rat photoreceptor cell apoptosis	Ganoderma spore lipid	2 ml/kg, once a day, 3 days before receiving 40 mg/kg dose of MNU	Apoptotic index (0h) = 0 ± 0 (%) Apoptotic index (1d) = 9.78 ± 1.26 (%) (p < 0.01 vs. NC, 0h) Apoptotic index (3d) = 21.88 ± 2.95 (%) (p < 0.01 vs. NC, 0h) Apoptotic index (7d) = 0.17 ± 0.05 (%) (p < 0.01 vs. 0h) Apoptotic index (10d) = 0 ± 0 (%)	By regulating the suppression of mouse photoreceptor cell death caused by MNU, G. lucidum spore lipids could protect retinal function
		PBS (Negative control)		Apoptotic index (0h) = 0 ± 0 (%) Apoptotic index (1d) = 18.30 ± 2.4 (%) (p < 0.01 vs. 0h) Apoptotic index (3d) = 60.63 ± 5.38 (%) (p < 0.01 vs. 0h) Apoptotic index (7d) = 0.25 ± 0.11 (%) (p < 0.01 vs. 0h) Apoptotic index (10d) = 0 ± 0 (%)	
Jin et al., (2013) [78]	Protect effectf on cadmium hepatotoxicity (n = 8)	Cd	3.7 mg/kg	Liver and body weight ratios = 58.53 ± 1.97 (mg/g) (p < 0.05 vs. control) serum ALT = 520.98 ± 38.04 (U/L) (p < 0.05 vs. control) serum AST = 1052.05 ± 76.71 (U/L) (p < 0.05 vs. control) Hepatic MT protein = 20.98 ± 0.98 (μg/g) (p < 0.05 vs. control)	The GLS effectively prevents hepatotoxicity brought on by Cd(II)
		GL	0.1 g/kg	Liver and body weight ratios = 57.03 ± 0.97 (mg/g) serum ALT = 450.73 ± 8.77 (U/L) serum AST = 947.95 ± 49.30 (U/L) Hepatic MT protein = 22.62 ± 2.29 (μg/g)	
			0.5 g/kg	Liver and body weight ratios = 53.97 ± 1.04 (mg/g) (p < 0.05 vs. Cd alone) serum ALT = 377.56 ± 11.71 (U/L) (p < 0.05 vs. Cd alone) serum AST = 805.48 ± 10.96 (U/L) (p < 0.05 vs. Cd alone) Hepatic MT protein = 31.15 ± 1.96 (μg/g) (p < 0.05 vs. Cd alone)	
			1.0 g/kg	Liver and body weight ratios = 52.06 ± 0.93 (mg/g) (p < 0.05 vs. Cd alone) serum ALT = 330.73 ± 5.85 (U/L) (p < 0.05 vs. Cd alone) serum AST = 745.21 ± 16.42 (U/L) (p < 0.05 vs. Cd alone) Hepatic MT protein = 41.97 ± 6.88 (μg/g) (p < 0.05 vs. Cd alone)	
Liu et al., (2021) [76]	Protective effect in trimethylamine-N-oxide induced cardiac dysfunction (n = 6)	Control		Cardiac output = 22.36 ± 1.54 (ml/mm) ^(*)^	XF can maintain the metabolic balance and function of the heart, and DT can reduce the risk of cardiovascular diseases
		Model		Cardiac output = 12.72 ± 0.88 (ml/mm) ^(*)^	
		DT	50 mg/kg/day	Cardiac output = 23.68 ± 1.1 (ml/mm) ^(*)^	
		XF	50 mg/kg/day	Cardiac output = 25.43 ± 1.32 (ml/mm) ^(*)^	
		ZF	50 mg/kg/day	Cardiac output = 20.17 ± 1.33 (ml/mm) ^(*)^	
Xie et al., (2016) [77]	Cardiovascular protective effect	Sham		LVEF = 65.23 (%) LVFS = 35.75 (%) Left ventricular end diastolic diameter = 3.83 (LV Trace, mm) Cardiac output = 20.37 (ml/min)	The ganoderma therapy restored the ejection fraction to normal and reversed the TAC-induced fractional shortening
		TAC + vegetable oil		LVEF = 43.26 (%) LVFS = 21.7 (%) Left ventricular end diastolic diameter = 4.63 (LV Trace, mm) Cardiac output = 20.28 (ml/min)	
		TAC + hypertesion drugs		LVEF = 53.27 (%) LVFS = 27.34 (%) Left ventricular end diastolic diameter = 4.21 (LV Trace, mm) Cardiac output = 21.3 (ml/min)	
		TAC + Ganoderma oil		LVEF = 66.02 (%) LVFS = 36.75 (%) Left ventricular end diastolic diameter = 4.01 (LV Trace, mm) Cardiac output = 24.1 (ml/min)	
Zhou et al., (2012) [80]	Neuroprotective effect in mice	Normal control		Neuron number = 2392.75 ± 90.63 ^(*)^	Pre-administration of H-GLS and M-GLS significantly reversed the number of neurons, same as control group
		Model control		Neuron number = 1314.2 ± 81.57 (significant difference vs. normal control) ^(*)^	
		H-GLS	8.0 g/kg	Neuron number = 2419.94 ± 72.51 (significant difference vs. model control) ^(*)^	
		M-GLS	4.0 g/kg	Neuron number = 2320.24 ± 81.57 (significant difference vs. model control) ^(*)^	
		L-GLS	2.0 g/kg	Neuron number = 1450.15 ± 72.51 ^(*)^	
Zhao et al., (2021) [93]	Efficiency on Alzheimer disease in mice (n = 8)	Vehicle control		BDNF = 98.71 ± 6.41 (%) TrkB = 99.99 ± 2.57 (%) pTrkB = 99.13 ± 7.83 (%) pTrkB /TrkB = 97.83 ± 9.13 (%)	Treatment with RGLS recovered the STZ-induced reductions in neurotrophic factors, including as BDNF, TrkB, and TrkB phosphorylation at Tyr 816
		STZ model		BDNF = 53.85 ± 6.41 (%) (p < 0.001 vs. control) TrkB = 48.72 ± 11.54 (%) (p < 0.001 vs. control) pTrkB = 23.48 ± 6.52 (%) (p < 0.001 vs. control) pTrkB/TrkB = 43.04 ± 6.52 (%) (p < 0.001 vs. control)	
		STZ + RGLS	180 mg/kg	BDNF = 69.23 ± 14.1 (%) TrkB = 64.1 ± 11.54 (%) pTrkB = 37.82 ± 11.75 (%) pTrkB/TrkB = 56.08 ± 9.13 (%)	
		STZ + RGLS	360 mg/kg	BDNF = 85.89 ± 11.55 (%) TrkB = 85.89 ± 8.98 (%) (p < 0.05 vs. STZ model) pTrkB = 60 ± 7.83 (%) (p < 0.01 vs. STZ model) pTrkB/TrkB = 73.04 ± 10.44 (%)	
		STZ + RGLS	720 mg/kg	BDNF = 116.66 ± 15.39 (%) (p < 0.01 vs. STZ model) TrkB = 94.87 ± 2.57 (%) (p < 0.01 vs. STZ model) pTrkB = 86.08 ± 6.52 (%) (p < 0.0001 vs. STZ model) pTrkB/TrkB = 89.99 ± 14.36 (%) (p < 0.05 vs. STZ model)	
Dai et al., (2019) [75]	Protection against radiation-induced heart disease in mice (n = 5)	GLSO@P188/PEG400 NS	3 ml/kg	Fibrosis area (Heart) = 11.49 ± 2.64 (%) (p < 0.01 vs. X-rays group) ^(*)^ Neorosis area (Ear) = 0.96 ± 0.23 (%) (p < 0.05 vs. X-rays group)^ (*)^ Neorosis area (Tail) = 1.52 ± 1.2 (%) (p < 0.01 vs. X-rays group)^ (*)^	pre- and post-treatment with GLSO@P188/PEG400 NS may protect the heart against X-rays
		Baseline group		Fibrosis area (Heart) = 1.17 ± 0.36 (%) ^(*)^ Neorosis area (Ear) = 0.22 ± 0.20 (%) ^(*)^ Neorosis area (Tail) = 0.92 ± 0.63 (%) ^(*)^	
		Sole X-rays (20 Gy) group		Fibrosis area (Heart) = 29.7 ± 2.64 (%) (p < 0.001 vs. baseline group) ^(*)^ Neorosis area (Ear) = 5.41 ± 0.63 (%) (p < 0.05 vs. baseline group) ^(*)^ Neorosis area (Tail) = 16.52 ± 2.43 (%) (p < 0.01 vs. baseline group) ^(*)^	
Jiao et al., (2020) [94]	Wound healing	GLSO		Collagen volume fraction (day 5) = 26.87 ± 7.87 (p < 0.01 vs. control)	GLSO significantly accelerated the healing of skin wounds compared to antibacterial therapy
Ge et al., (2009) [67]	Effects on sialoadenitis in mice (n = 8)	High-dose GLS	1.0 g/kg/day	CD3+T = 74.56 ± 7.56 CD4+/CD8+ = 2.83 ± 0.69 (p < 0.05 vs control) CD4+T apoptosis = 31.12 ± 6.37 (p < 0.05 vs control) CD19+B apoposis = 9.21 ± 4.19 (p < 0.05 vs control) IgG = 162.59 ± 43.35 (μg/ml) (p < 0.05 vs control)	The ratio of CD4+/CD8+ T lymphocytes and the serum IgG levels of NOD mice dramatically reduced after pretreatment with H-GLS prior to the start of sialoadenitis
		Normal saline (NS) control	0.2 ml	CD3+ T = 68.81 ± 12.57 CD4+/CD8+ = 5.44 ± 0.4 CD4+ T apoptosis = 36.08 ± 14.58 IgG = 200.76 ± 38.15 (μg/ml) CD19+ B apoptosis = 10.04 ± 3.46	
Clinical trial					
Deng et al., (2021) [64]	Immunological activity in post‑operative breast and lung cancer patients	GLS powder (n = 63)		CD3+ = 72 ± 6 (p < 0.01 vs. control) CD3+ CD4+ = 42 ± 6.4 (p < 0.05 vs. control) CD3+ CD16+ CD56+ = 12.5 ± 6 (p < 0.01 vs. control) CD4+ CD25+ = 8.4 ± 3.5 (p < 0.05 vs. control) CD3+ HLADR+ = 1.7 ± 1 (p < 0.01 vs. control) CD3+ HLADR = 70.4 ± 5.6 (p < 0.01 vs. control) CD4+ HLADR+ = 1.9 ± 1 (p < 0.01 vs. control) CD4+ HLADR− = 41.9 ± 6.8 (p < 0.01 vs. control) CD8+ HLADR+ = 0.7 ± 0.5 (p < 0.01 vs. control) CD8+ HLADR− = 28.2 ± 6.8 (p < 0.05 vs. control)	Patients who are most likely to benefit from the immunological improvements brought on by G. lucidum therapy may be identified through T lymphocyte subsets in combination with pertinent cytokines and AGR/NLR inflammatory predictors
		Control (n = 57)		CD3+ = 66.4 ± 10.6 CD3+ CD4+ = 37.7 ± 10.5 CD3+ CD16+ CD56+ = 16.9 ± 11.0 CD4+ CD25+ = 10.0 ± 4.0 CD3+ HLADR+ = 9.7 ± 6.5 CD3+ HLADR = 56.3 ± 12.5 CD4+ HLADR+ = 3.5 ± 2.4 CD4+ HLADR− = 37.0 ± 10.8 CD8+ HLADR+ = 5.3 ± 5.0 CD8+ HLADR− = 24.9 ± 8.0	
Wang et al., (2018) [99]	Epilepsy treatment in patient (n = 18)	Before treatment		Weekly seizure frequency = 3.1 ± 0.8 QOLIE-31 = 55.8 ± 7.5 Each seizure episode = 12.8 ± 5.1 (min)	GLSP may be helpful in lowering the frequency of weekly seizures
		After treatment (GLSP, 1000 mg each time; 3 times daily for 8 weeks)		Weekly seizure frequency = 2.4 ± 1.2 (p = 0.04) QOLIE-31 = 60.4 ± 9.6 (p = 0.11) Each seizure episode = 15.3 ± 4.8 (min) (p = 0.13)	
Zhao et al., (2012) [47]	Improves cancer-related fatigue in breast cancer patients undergoing endocrine therapy	Control (n = 23)		TNF-α = 131.21 ± 16.52 TNF-α 4 weeks = 127.43 ± 16.52 IL-6 = 66.26 ± 10.06 IL-6 4 weeks = 64.05 ± 10.31	GLS powder may improve quality of life and reduce tiredness associated with cancer in breast cancer patients receiving endocrine treatment
		Experiment (G. lucidum 1000 mg three times a day for 4 weeks) (n = 25)		TNF-α = 128.37 ± 16.05 (p < 0.01 vs. control) TNF-α 4 weeks = 71.74 ± 15.58 (p < 0.01 vs. control) IL-6 = 62.09 ± 8.58 (p < 0.05 vs. control) IL-6 4 weeks = 41.47 ± 8.1 (p < 0.05 vs. control)	

Safety

­No serious side effects were reported and there were no abnormalities in liver or kidney function when *G. lucidum* spore powder was used in patients [45,47,64]. Stomach discomfort, nausea, vomiting, fatigue, dizziness, dry mouth, colitis or diarrhea, epistaxis, and sore throat are among the adverse events reported [47,64,99].

However, current data show that cancer patients using *G. lucidum* spore powder have abnormally elevated serum CA72-4 levels. Monitoring of CA72-4 levels may be necessary when using *G. lucidum* spore powder to monitor the decision of whether to discontinue use or not [46,101].

Risk-of-Bias of Included Studies

Among the in vitro studies, 27 studies were considered low risk of bias, nine studies had a moderate risk of bias, four studies had a high risk of bias, and none were excluded due to quality. All in vivo studies are considered to have a moderate risk of bias because many domains do not have enough detailed information reported to accurately assess the risk of bias. A retrospective study is of fair quality, a case report is of good quality, and a case report is of fair quality. Three clinical trials had a moderate risk of bias. See Appendix 2-7 for the details. A summarized quality assessment of all included studies is presented in Table 3.

**Table 3:** Summarized quality assessment of all included studies

Study	Conclusion
Fukuzawa et al., 2008 [12]	low
Gao et al., 2012 [13]	low
Xinlin et al., 1997 [37]	moderate
Lu et al., 2004 [14]	moderate
Lu et al., 2004 [15]	low
Oliveira et al., 2014 [16]	low
Sliva et al., 2002 [19]	high
Sliva et al., 2003 [20]	low
Song et al., 2021 [33]	low
Wang et al., 2019 [21]	low
Zhong et al., 2021 [40]	low
Zhu et al., 2000 [30]	high
Wu et al., 2012 [43]	low
Li et al., 2016 [32]	moderate
Chan et al., 2005 [51]	moderate
Chan et al., 2007 [52]	low
Hsu et al., 2012 [55]	low
Ma et al., 2008 [53]	moderate
Zhang et al., 2011 [50]	moderate
Cai et al., 2021 [65]	low
Saavedra Plazas et al., 2020 [69]	low
Nguyen and Nguyen, 2015 [71]	high
Shen et al., 2019 [68]	low
Heleno et al., 2012 [70]	moderate
Nayak et al., 2021 [84]	low
Nayak et al., 2015 [85]	low
Nayak et al., 2010 [83]	high
Shen et al., 2020 [18]	low
Zhu et al., 2018 [86]	low
Zhu et al., 2019 [31]	low
Yang et al., 2020 [92]	low
Wang et al., 2012 [17]	low
Wang et al., 2014 [82]	low
Pan et al., 2019 [81]	low
Weng et al., 2010 [100]	moderate
Huang et al., 2011 [95]	low
Li et al., 2013 [96]	low
Wang et al., 2013 [97]	low
Yang et al., 2016 [98]	moderate
Li et al., 2020 [79]	moderate
Chen et al., 2016 [41]	moderate
Chen et al., 2016 [36]	low
Dai et al., 2021 [44]	low
Jiao et al., 2020 [42]	moderate
Li et al., 2017 [35]	moderate
Na et al., 2017 [26]	moderate
Shi et al., 2021 [39]	moderate
Su et al., 2018 [23]	moderate
Su et al., 2018 [28]	moderate
Zhang et al., 2019 [25]	moderate
Pan et al., 2019 [27]	moderate
Wang et al., 2012 [29]	moderate
He et al., 2020 [24]	moderate
Guo et al., 2009 [54]	moderate
Yue et al., 2008 [38]	moderate
Bao et al., 2002 [48]	moderate
Bao et al., 2001 [49]	moderate
Dai et al., 2019 [75]	moderate
Fu et al., 2019 [34]	moderate
Liu et al., 2002 [22]	moderate
Bao et al., 2001 [56]	moderate
Bao et al., 2001 [57]	moderate
Li et al., 2020 [61]	moderate
Liu et al., 2021 [59]	moderate
Su et al., 2021 [58]	moderate
Wang et al., 2017 [62]	moderate
Wu et al., 2020 [60]	moderate
Ma et al., 2009 [63]	moderate
Sang et al., 2021 [66]	moderate
Levin et al., 2017 [72]	moderate
Zhang et al., 2021 [73]	moderate
Zhan et al., 2016 [87]	moderate
Jiang et al., 2021 [88]	moderate
Lai et al., 2020 [91]	moderate
Shaher et al., 2020 [89]	moderate
Wang et al., 2015 [90]	moderate
Gao et al., 2010 [74]	moderate
Jin et al., 2013 [78]	moderate
Liu et al., 2021 [76]	moderate
Xie et al., 2016 [77]	moderate
Zhou et al., 2012 [80]	moderate
Zhao et al., 2021 [93]	moderate
Jiao et al., 2020 [94]	moderate
Ge et al., 2009 [67]	moderate
Wang et al., 2018 [99]	moderate
Liang et al., 2013 [101]	low
Yan et al., 2014 [46]	moderate
Suprasert et al., 2013 [45]	moderate
Deng et al., 2021 [64]	moderate
Zhao et al., 2012 [47]	moderate

Discussion

In general,* G. lucidum* spores possess ingredients that are very similar to other parts of *G. lucidum*, although spores contain a higher concentration of some bioactive compounds [3,102]. However, to the best of our knowledge, there is no article to date comparing the efficiency between extracts of different parts thus establishing the need for such investigations to identify the benefits of *G. lucidum* spores over its other parts.

*G. lucidum* spores and the extract from the spores both show effective anti-tumor, immunomodulatory, anti-inflammatory, and antioxidant activities in treatment and in research. The comparison between UBSG and BSG showed that the effects of BSG were greater than those of the UBSG [30,37,38]. The phytochemical experiment showed that BSG contained higher contents of total carbohydrates and amino acids than UBSG. Triterpenes and polysaccharides from *G. lucidum* were well-known for its significant anticancer activity and immunomodulation [3,102]. This could be an explanation for the stronger effects of BSG compared to UBSG. In addition, the purification of BSG extract by chromatography revealed even more remarkable anti-tumor activities. This suggested that the purified extract might possess compounds that were responsible for the effect. However, to our knowledge, no significant studies have taken place to explore ingredients in such fractions to confirm this hypothesis. We suggest further studies screening potential compounds of purified BSG extract.

Besides, our research also realized that alcohol extracts and aqueous extracts have different therapeutic effects and effects in different areas of study. Namely, BSGEE showed a stronger inhibitory effect on tumors than BSGWE, while BSGWE had a stronger efficacy on immune systems. Previously, it was estimated that BSGEE had triterpenes whereas BSGWE had polysaccharides as major content [3,102,103]. This could imply that triterpenes play a critical role in anti-tumor activities while polysaccarides show better modulation of the immune system. BSGEE showed its cytotoxic activity via arresting G1 phase of cell cycle meanwhile ethanol/ethanol BSG extract blocked G2/M phase [30,36]. It appeared that the ethanol/ethanol fraction possessed bioactive substances different from ethanol extract. Phytochemical experiments should be conducted in the future for clarification. 

There is also evidence of antimicrobial activitiesof *G. lucidum* spore, even on resistant bacteria, via MIC results. Extracts were considered highly active against bacteria when MIC < 100 µg/ml [104]. Thus, BSGWE could be deemed to possess antibacterial activity against *Enterococcus faecalis* and *K. pneumoniae* as the MIC values are 2-62.5 µg/ml. Moreover, the effect on the metabolites of *G. lucidum* spore contributes to alleviating the severity of chronic diseases through hypoglycemic and hypolipidemic activities. The modulation of body metabolism is possibly activated via GS2 and GYG1 genes (involved in glycogen synthesis), Insig1 and Insig2 genes (involved in glucose homeostasis and cholesterol homeostasis), Acox1 gene (involved in lipid oxidation), and ACC and Fads1 genes (involved in lipogenesis suppression). Additionally, Lai et al. also demonstrated that *BSGEE* inhibited lipid levels via the upregulation of LXRα expression leading to the increase in downstream genes such as ABCA1 and ABCG1. Thus, cholesterol molecules were transported back to the liver resulting in a decrease in blood cholesterol.

*G. lucidum* spore also has a supportive effect in the treatment of Alzheimer's disease treatment, anti-aging, wound healing, proliferation enhancer, and epilepsy treatment. The Aβ level and Tau phosphorylation excess are known for being associated with Alzheimer's disease [105]. Therefore, the suppression of Aβ level and Tau phosphorylation caused by *G. lucidum* spore extract could explain its potential against Alzheimer's disease. However, the concentrations of extract used in this experiment were quite high (up to 720 mg/kg) and the difference in the number of crossings to the platform location in the Morris water maze test across groups was not significant [93]. Consequently, we suggest further studies to confirm the benefits of *G. lucidum* spore extract to prevention and treatment of Alzheimer's disease.

Furthermore, the safety of *G. lucidum* spore is noteworthy, as no anomalies of bodily organs have been documented. Nevertheless, caution must be exercised when administering it to cancer patients, given the lack of adequately reported selectivity index values on varied cancer cells. Moreover, rigorous monitoring of patients is vital when administering a total daily dose of 1800 mg (or taken as two separate doses of 900 mg each per day), due to the potential occurrence of adverse events associated with this dosage.

The characteristics of the included studies are given in Table 4.

**Table 4:** Baseline characteristics of included studies

Author (Year)	Study design	Intervention	Pharmacological activities	Out come
Fukuzawa et al., (2008) [12]	in vitro	Long chain fatty acids in the spores	Antitumor activity	IC_50_ (µM), TNF-α release (pg/ml), HL-60 growth (% of control)
Gao et al., (2012) [13]	in vitro	C-19 fatty acids	Antitumor activity	Apoptotic cells
Xinlin et al., (1997) [37]	in vitro	Sporoderm-broken spores of G. lucidum (BSG), sporoderm-nonbroken spores of G. lucidum (NBSG)	Antitumor activity	OD value
Lu et al., (2004) [14]	in vitro	Extraction of G. lucidum spore powder	Antitumor activity	Cell proliferation (%)
Lu et al., (2004) [15]	in vitro	Extraction of G. lucidum spore powder	Antitumor activity	Cell proliferation (%)
Oliveira et al., (2014) [16]	in vitro	Phenolic extraction of G. lucidum spore	Antitumor activity	GI_50_ (µg/mL)
Sliva et al., (2002) [19]	in vitro	G. lucidum spores	Antitumor activity	Migration (%), relative NF-kB activity (%), relative AP-1 activity (%)
Sliva et al., (2003) [20]	in vitro	G. lucidum spores	Antitumor activity	Migration (%), relative NF-kB activity (%)
Song et al., (2021) [33]	in vitro	Ganoderma lucidum spore powder	Antitumor activity	OD, inhibiton rate (%), cell (%), apoptosis (%), TNF-α levels (pg/ml), IL-1β levels (pg/ml), IL-6 levels (pg/ml), TGF-β1 levels (pg/ml)
Wang et al., (2019) [21]	in vitro	Extract prepared from G lucidum spores	Mediated immunomodulation and cancer treatment	Fold change in PD -1 protein, % of PD-1 cells, fold change in CCL5 prtotein
Zhong et al., (2021) [40]	in vitro	Polysaccharides from RSGand BSG	Antitumor activity	IC_50_, cell apoptosis rate (%)
Zhu et al., (2000) [30]	in vitro	Extracts from BSG	Antitumor activity	Death ratio (%), IC_50_
Wu et al., (2012) [43]	in vitro	Ganoderma oil	Antitumor activity	Cell number, EC_50_, cell survival
Li et al., (2016) [32]	in vitro	Supercritical-CO2 extraction	Inhibits cholangiocarcinoma cell migration	Cell viability (%), number of cell migration
Chan et al., (2005) [51]	in vitro	Extract of . lucidum spore	Immunological activity	Relative cell proliferation (%)
Chan et al., (2007) [52]	in vitro	Crude spore polysaccharides (GL-S), pure spore polysaccharides (GL-SG)	Immunological activity	Relative cell proliferation (%), IL-10 (pg/mL)
Hsu et al., (2012) [55]	in vitro	G. lucidum spores extract	Immunological activity	Phagocytic activity of human polymorphonuclear neutrophils (mean fluorescence intensity %)
Ma et al., (2008) [53]	in vitro	Polysaccharides from Ganoderma lucidum spores	Immunological activity	Cell proliferation (fold of control), IL-2 production, TNF-α production
Zhang et al., (2011) [50]	in vitro	Water-soluble polysaccharide of Ganoderma lucidum spores	Immunological activity	Murine lymphocyte proliferation index (A570)
Cai et al., (2021) [65]	in vitro	Water extract, alcohol extract of sporoderm-removed Ganoderma lucidum spores (SR-GLS)	Anti-inflammatory	Indicator A (acetic acid - propionic acid - butyric acid)/total short-chain fatty acids; indicator B (isobutyric acid + isovaleric acid)
Saavedra Plazas et al., (2020) [69]	in vitro	RM, BR, MBR1	Antioxidant activity	% inhibition DPPH (%)
Nguyen and Nguyen (2015) [71]	in vitro	G. lucidum spore powder	Antioxidant activity	Antioxidant activity
Shen et al., (2019) [68]	in vitro	Ganoderma lucidum spore powder	Antioxidant activity, improves glucose consumption in insulin-resistant HepG2 cells	% inhibition DPPH (%), glucose consumption (mmol/L)
Heleno et al., (2012) [70]	in vitro	Phenolic and polysaccharidic extracts	Antioxidant activity	DPPH scavenging activity (mg/ml), reducing power (mg/ml), β-carotene bleaching inhibition (mg/ml), EC_50_ (mg/ml)
Nayak et al., (2021) [84]	in vitro	Ganoderma lucidum spores	Antimicrobial activity	Minimum inhibitory concentration value (mcg/ml)
Nayak et al., (2015) [85]	in vitro	Spore of Ganoderma lucidum	Antimicrobial activity	Percentage of sensitive (%), percentage of resistant (%)
Nayak et al., (2010) [83]	in vitro	Spore of Ganoderma lucidum	Antimicrobial activity	Minimum inhibitory concentration value (mcg/ml)
Shen et al., (2020) [18]	in vitro	Triterpenoid extracts from Ganoderma lucidum spore powder	Antibacterial, antioxidant and anti-cancer	Average inhibition zone diameter (mm), DPPH radical-scavenging activities (%), cell viability (%)
Zhu et al., (2018) [86]	in vitro	Chitosan from Ganoderma lucidum spore powder	Antimicrobial activity	Average inhibition zone diameter (mm)
Zhu et al., (2019) [31]	in vitro	Proteoglycan from cracked (proteoglycan-C) and uncracked Ganoderma lucidum spore powder (proteoglycan-UC)	Antimicrobial, hyperglycemic, antitumor and antioxidant	Average inhibition zone diameter (mm), DPPH radical-scavenging activities (%), cell viability (%), glucose concentration (mmol/L)
Yang et al., (2020) [92]	in vitro	Oligosaccharide from spores of Ganoderma lucidum	Prebiotic effects	Growth rate of Lactobacillus acidophilus
Li et al., (2020) [79]	in vitro	Sporoderm-broken spore of G. lucidum	Induced intestinal barrier injury	Apoptosis (%)
Wang et al., (2012) [17]	in vitro	Ganoderma lucidum spores	Induced apoptosis in human leukemia THP-1 cells	Apotosis rate (%)
Wang et al., (2014) [82]	in vitro	Ganoderma lucidum spores	Inhibitive effect on apoptosis	Apoptotic rate (TUNEL) (%), splenic index (mg/g)
Pan et al., (2019) [81]	in vitro	Ganoderma spore lipid	Protects bone marrow mesenchymal stem cells and hematopoiesis	Apoptosis rate, erythrocyte colony forming unit (CFU-E), erythroid burst-forming units (BFU-E), granulocyte macrophage colony-forming units (CFU-GM)
Huang et al., (2011) [95]	in vitro	Ganoderma lucidum spore lipid	Induced the activity of PPARα	PPARα fold induction
Li et al., (2013) [96]	in vitro	Ganoderma lucidum spore	Enhance of embryonic stem cells	Specific growth rate (%)
Wang et al., (2013) [97]	in vitro	Ganoderma lucidum spore	Anti-epileptic effects	Fluorescent intensity values, the expression level of NT-4, the expression level of N-cadherin
Yang et al., (2016) [98]	in vitro	Ganoderma lucidum spore	Anti-epileptic effects	BDNF fluorescence intensity, TRPC3 fluorescence intensity, apoptosis rate
Chen et al., (2016) [41]	in vitro, in vivo	Ganoderma spores oil	Antitumor effect	Half maximal inhibitory concentration (IC_50_), inhibitory rate (%)
Chen et al., (2016) [36]	in vitro, in vivo	E/E-SBGS (Ethanol/ethanol extract () from SBGS (Ganoderma lucidum sporoderm-broken spores) ()	Antitumor effect	Migration of lung cancer cells (H441 cells) (% of control), colony number (% of control), tumor volume (mm^3^), tumor weight (g)
Dai et al., (2021) [44]	in vitro, in vivo	G.lucidum spore oil (GLSO) nanosystems (GLSO@NEs)	Antitumor effect	Half maximal inhibitory concentration (IC_50_), apoptosis analysis (MGC803 cells) (%), migrated cell (% of control), invaded cell (% of control), tumor volume (mm^3^), tumor weight (g)
Jiao et al., (2020) [42]	in vitro, in vivo	G. lucidum spore oil	Antitumor effect	Fold change of control, % apoptosis area
Li et al., (2017) [35]	in vitro, in vivo	Ethanol extracts of BSGLEE (G. lucidum sporoderm-broken spores)()	Antitumor effect	Cell viability (% of control), cell cycle distribution (%), apoptosis (%), average migration cells, tumor weight (g), liver weight (g)
Na et al., (2017) [26]	in vitro, in vivo	G. lucidum sporoderm-broken spores water extract (BSGLWE)	Anticarcinogenic effects	Cell viability (%), tumor weight (g)
Shi et al., (2021) [39]	in vitro, in vivo	Ganoderma lucidum spore (GLS), wall-broken Ganoderma lucidum powder (BGLSP) and wall-removed Ganoderma lucidum powder (RGLSP)	Antitumor effect	IC_50_, inhibition rate (%)
Su et al., (2018) [23]	in vitro, in vivo	Sporoderm-breaking spores of G. lucidum	Antitumor effect	Cell viability (%), tumor volume (mm^3^), tumor weight (g)
Su et al., (2018) [28]	in vitro, in vivo	BSGLP (polysaccharide of the G. lucidum sporoderm-breaking spores)	Antitumor effect	Tumor, IOD/10^6^ pixel
Zhang et al., (2019) [25]	in vitro, in vivo	BSGLWE (Water extract of Ganoderma lucidum sporoderm-broken spores)	Antitumor effect	Cell viability (%), apoptotic cells (%), tumor volume (mm^3^), tumor weight (g)
Pan et al., (2019) [27]	in vitro, in vivo	Polysaccharides from Ganoderma lucidum sporoderm-broken spores	Antitumor effect	Cell viability (%), tumor volume (mm^3^), tumor weight (g)
Wang et al., (2012) [29]	in vitro, in vivo	BSGLP (Polysaccharides from Ganoderma lucidum broken-spore)	Immunological activity, antitumor effect	Inhibitory ratio, proliferation ratio, CD4+/CD8+
He et al., (2020) [24]	in vitro, in vivo	BSGLWE (Water extract of Ganoderma lucidum sporoderm-broken spores)	Immunological activity, antitumor effect	Apoptosis rate (%), STAT3, pho-STAT3, tumor volume (mm^3^)
Guo et al., (2009) [54]	in vitro, in vivo	G. lucidum spore polysaccharide	Immunological activity, antitumor effect	TNF-α and IL-6 secretion (pg/mL), Tumor weight (g)
Yue et al., (2008) [38]	in vitro, in vivo	sporoderm-broken Ganoderma spores and sporoderm -unbroken Ganoderma spores	Immunological activity, antitumor effect	TNF-α and IL-6 secretion (pg/mL), cell proliferation (%), tumor weight (g)
Bao et al., (2002) [48]	in vitro, in vivo	PSGL-I-1A	Immunological activity	T lymphocytes proliferation index (A570)
Bao et al., (2001) [49]	in vitro, in vivo	G. lucidum spore polysaccharide (PSG)	Immunological activity	B and T lymphocytes proliferation index (A570)
Dai et al., (2019) [75]	in vitro, in vivo	Ganoderma lucidum spore oil (5mL) @P188/PEG400 nanosystem (GLSO@P188/PEG400 NS)	Protection against radiation-induced heart disease	Cell viability (% of control), Relative intensity of phosphorylated γ-H2A.X (fold change), Fibrosis area (%), Neorosis area (%)
Fu et al., (2019) [34]	in vivo	GLSP (Polysaccharide from Ganoderma lucidum spores)	Antitumor effect	Tumor weight (g)
Liu et al., (2002) [22]	in vivo	Sporoderm-broken germinating Ganoderma lucidum spores	Antitumor effect	Tumor weight (g)
Bao et al., (2001) [56]	in vivo	Glucans from spore G. lucidum (PGL)	Immunological activity	B and T lymphocytes proliferation index (A570), antibody production (A520)
Bao et al., (2001) [57]	in vivo	Native polysaccharide (SP) and the Smith-degraded polymer of the SP (SP-1)	Immunological activity	B and T lymphocytes proliferation index (A570), antibody production (A520), serum IgG, complement (C-3) levels
Li et al., (2020) [61]	in vivo	Sporoderm-broken of Ganoderma lucidum spores (BGLS), sporoderm-removed Ganoderma lucidum spores Ganoderma lucidum spores (RGLS)	Immunological activity	The number of neutrophils, neutrophil recovery rate (%), the number of macrophage that phagocytized ACNP, macrophage formation efficiency, macrophage phagocytosis efficiency
Liu et al., (2021) [59]	in vivo	Water extracts from unbroken spores of Ganoderma lucidum	Immunological activity	Serum half-hemolytic value (HC_50_)
Su et al., (2021) [58]	in vivo	Polysaccharide of spores of G. lucidum	Immunological activity	Thymus coeficiency, NK cell’s tumor-killing ability
Wang et al., (2017) [62]	in vivo	Water soluble β-glucan (GLSWA-I)	Immunological activity	Ear swelling (mg)
Wu et al., (2020) [60]	in vivo	Spore oil of G. lucidum (GLSO)	Immunological activity	Phagocytic index, NK activity
Ma et al., (2009) [63]	in vivo	Ganoderma lucidum spore polysaccharides	Immunological activity, against cyclophosphamide (Cy) toxicity	Thymus weight (mg), Con-A induced lymphocyte proliferation
Sang et al., (2021) [66]	in vivo	BGLSP (Polysaccharide of Ganoderma lucidum sporoderm-broken spore)	Anti-inflammatory, anti-obesity	Body weight gain (g), TC (mmol/L), LDL (mmol/L), TG (mmol/L), HDL (mmol/L), NEFA (mmol/L), TNF-α (ng/L), IL-1β (ng/L), IL-6 (ng/L), MCP-1 (ng/L), Positive area (%)
Levin et al., (2017) [72]	in vivo	G. lucidum broken spore shell extracts	Protection of bladder function following oxidative stress	Bladder weight (mg), Compliance (cm H_2_O/20% capacity)
Zhang et al., (2021) [73]	in vivo	Ganoderma lucidum spore oil (GLSO)	Antioxidant activity	Life span in the condition of oxidative stress
Zhan et al., (2016) [87]	in vivo	Ganoderma lucidum extract (spores andspores lipid)	Antimicrobial activity	LogCFU
Jiang et al., (2021) [88]	in vivo	Resistant starch encapsulated Ganoderma lucidum spores (EGLS)	Glucose/lipid metabolism and gut microbiota	Blood glucose concentration, total cholesterol (TC), triglyceride (TG) and high-density lipoprotein cholesterol (HDL-C) levels
Lai et al., (2020) [91]	in vivo	Ganoderma lucidum spore ethanol extract (EEG)	Lipid-lowering and anti-atherosclerotic effects	Total cholesterol/high-density lipoprotein cholesterol (TC/HDL-C) ratio, aterial intima/medium thickness (I/M), hepatocyte steatosis (score)
Shaher et al., (2020) [89]	in vivo	Ganoderma lucidum spores (GLS)	Hyperglycemia-mediated cardiomyopathy protection	Body weight (g), blood glucose, HbA1C, BNP/GAPDH, TNF-α/GAPDH, IL-1β/GAPDH, Caspase-3/GAPDH
Wang et al., (2015) [90]	in vivo	Ganoderma lucidum spores powder (GLSP)	Glucose and lipid metabolisms	Blood glucose level (mmol/L), TG (mmol/L), HDL-C (mmol/L)
Gao et al., (2010) [74]	in vivo	Ganoderma spore lipid	Protecting retinal function against N-methyl-N-nitrosourea	Apoptotic index (%)
Jin et al., (2013) [78]	in vivo	Ganoderma lucidum spores	Protect effectf on cadmium hepatotoxicity	Liver and body weight ratios (mg/g), serum ALT (U/L), serum AST (U/L), hepatic MDA (nmol/g liver), hepatic MT protein (μg/g)
Liu et al., (2021) [76]	in vivo	Extract from spores of Ganoderma lucidum	Protective effect in trimethylamine-N-oxide induced cardiac dysfunction	Ejection fraction, fractional shortening, cardiac output, content of TMAO
Xie et al., (2016) [77]	in vivo	Ganoderma spore oil	Cardiovascular protective effect	Left ventricular ejection fraction - LVEF (%), left ventricular fractional shortening - LVFS (%), left ventricular end diastolic diameter (LV Trace, mm), cardiac output (ml/min)
Zhou et al., (2012) [80]	in vivo	Ganoderma lucidum spores	Neuroprotective effect	GSH index (mg/g pr), GR index (U/g Pr), MDA index (nmol/mg.PR), CytOx (U/mcg min), ATP (mcg/ml), neuron number
Zhao et al., (2021) [93]	in vivo	Sporoderm-deficient Ganoderma lucidum spores (RGLS)	Efficiency on Alzheimer disease	BDNF (%), TrkB (%), pTrkB (%), pTrkB/TrkB (%)
Jiao et al., (2020) [94]	in vivo	Ganoderma lucidum spore oil	Wound healing	Collagen volume fraction, area fraction (CD4), area fraction (CD8), area fraction (CD45), area fraction (IFN-γ), fold change of control (IL-4)
Ge et al., (2009) [67]	in vivo	Ganoderma lucidum spores	Effects on sialoadenitis	Incidence (μm^2^), Area, CD3+T, CD4+/CD8+, CD4+T apoptosis, CD8+T apoptosis, CD19+B, CD19+B apoposis, IgG (μg/ml)
Deng et al., (2021) [64]	Clinical trial	G. lucidum spore powder	Immunological activity	Detection results of T cell subsets
Wang et al., (2018) [99]	Retrospective study	Ganoderma lucidum spore powder (GLSP)	Epilepsy treatment	Weekly seizure frequency after, QOLIE-31, each seizure episode (min)
Liang et al., (2013) [101]	Case report	Ganoderma lucidum spore powder (GLSP)	Safety	CA72-4 levels
Weng et al., (2010) [100]	in vitro	Ganodermasides A and B	anti-aging	Cell viability (%)
Suprasert et al., (2013) [45]	Randomized double blind controlled trial	Spores lingzhi	Effect in cancer patients	Clinical characteristics
Yan et al., (2014) [46]	Case report	Spore of Ganoderma lucidum (GLS)	Induced CA72-4 elevation in gastrointestinal cancer	CA72-4 Values
Zhao et al., (2012) [47]	A pilot clinical trial	Spore powder of Ganoderma lucidum	Improves cancer-related fatigue in breast cancer patients undergoing endocrine therapy	TNF-α, IL-6

Limitations

Our limitation in this review was the language criteria. There are many reports on the biological effects of *G. lucidum* spore written in Chinese. The exclusion of these articles may cause certain shortcomings when compiling information about the therapeutic capabilities of *G. lucidum* spore. Nevertheless, our study included a large number of relevant articles, thus, the review appeared to relatively sufficiently summerize bioactivities of *G. lucidum* spore. In addition, unique compounds of *G. lucidum* spores have not been studied for their pharmacological effects yet. Therefore, we recommend further studies conducting experiments on these compounds. This could contribute to a deeper understanding of the pharmacological characteristics of *G. lucidum* spore, which will help in developing new materials for treating diseases.

## Conclusions

*G. lucidum *spore and its extracts have a lot of pharmacological potentials which may yield new approaches to treatments. Anti-tumor, immunomodulatory, anti-inflammatory, and antioxidant activities are the main effects of *G. lucidum* spore extracts. Sporoderm breaking technique could contribute to the production of extracts with more effective prevention and treatment of diseases. In addition, the potential of* G. lucidum* spore extract on Alzheimer’s disease should be tested. High doses of *G. lucidum* spore extract must be used with caution as there was a concern about the increase in cancer antigens.

## Appendices

Appendix 1

**Table 5:** PRISMA Checklist PRISMA: Preferred Reporting Items for Systematic Reviews and Meta-Analyses

Section/topic	#	Checklist item	Reported on page #	
TITLE				
Title	1	Identify the report as a systematic review, meta-analysis, or both.	1	
Structured summary	2	Provide a structured summary including, as applicable: background; objectives; data sources; study eligibility criteria, participants, and interventions; study appraisal and synthesis methods; results; limitations; conclusions and implications of key findings; systematic review registration number.	3	
Rationale	3	Describe the rationale for the review in the context of what is already known.	4	
Objectives	4	Provide an explicit statement of questions being addressed with reference to participants, interventions, comparisons, outcomes, and study design (PICOS).	4	
Protocol and registration	5	Indicate if a review protocol exists, if and where it can be accessed (e.g., Web address), and, if available, provide registration information including registration number.	5	
Eligibility criteria	6	Specify study characteristics (e.g., PICOS, length of follow-up) and report characteristics (e.g., years considered, language, publication status) used as criteria for eligibility, giving rationale.	5	
Information sources	7	Describe all information sources (e.g., databases with dates of coverage, contact with study authors to identify additional studies) in the search and date last searched.	5	
Search	8	Present full electronic search strategy for at least one database, including any limits used, such that it could be repeated.	5	
Study selection	9	State the process for selecting studies (i.e., screening, eligibility, included in systematic review, and, if applicable, included in the meta-analysis).	5	
Data collection process	10	Describe method of data extraction from reports (e.g., piloted forms, independently, in duplicate) and any processes for obtaining and confirming data from investigators.	6	
Data items	11	List and define all variables for which data were sought (e.g., PICOS, funding sources) and any assumptions and simplifications made.	N/A	
Risk of bias in individual studies	12	Describe methods used for assessing risk of bias of individual studies (including specification of whether this was done at the study or outcome level), and how this information is to be used in any data synthesis.	6	
Summary measures	13	State the principal summary measures (e.g., risk ratio, difference in means).	N/A	
Synthesis of results	14	Describe the methods of handling data and combining results of studies, if done, including measures of consistency (e.g., I^2^) for each meta-analysis.	N/A	
Risk of bias across studies	15	Specify any assessment of risk of bias that may affect the cumulative evidence (e.g., publication bias, selective reporting within studies).	N/A	
Additional analyses	16	Describe methods of additional analyses (e.g., sensitivity or subgroup analyses, meta-regression), if done, indicating which were pre-specified.	N/A	
RESULTS				
Study selection	17	Give numbers of studies screened, assessed for eligibility, and included in the review, with reasons for exclusions at each stage, ideally with a flow diagram.	6	
Study characteristics	18	For each study, present characteristics for which data were extracted (e.g., study size, PICOS, follow-up period) and provide the citations.	6	
Risk of bias within studies	19	Present data on risk of bias of each study and, if available, any outcome level assessment (see item 12).	9	
Results of individual studies	20	For all outcomes considered (benefits or harms), present, for each study: (a) simple summary data for each intervention group (b) effect estimates and confidence intervals, ideally with a forest plot.	7-9	
Synthesis of results	21	Present results of each meta-analysis done, including confidence intervals and measures of consistency.	N/A	
Risk of bias across studies	22	Present results of any assessment of risk of bias across studies (see Item 15).	N/A	
Additional analysis	23	Give results of additional analyses, if done (e.g., sensitivity or subgroup analyses, meta-regression [see Item 16]).	N/A	
DISCUSSION				
Summary of evidence	24	Summarize the main findings including the strength of evidence for each main outcome; consider their relevance to key groups (e.g., healthcare providers, users, and policy makers).	10	
Limitations	25	Discuss limitations at study and outcome level (e.g., risk of bias), and at review-level (e.g., incomplete retrieval of identified research, reporting bias).	12	
Conclusions	26	Provide a general interpretation of the results in the context of other evidence, and implications for future research.	10-12	
FUNDING				
Funding	27	Describe sources of funding for the systematic review and other support (e.g., supply of data); role of funders for the systematic review.	N/A	

Appendix 2

**Table 6:** Quality assessment of in vitro studies according to the items of the Modified CONSORT checklist CONSORT: Consolidated Standards of Reporting Trials

Study	Abstract	Scientific background and explanation of rationale?	Specific objectives and/or hypotheses?	Intervention	Outcomes	Sample size	Randomization - Sequence generation	Randomization - Allocation concealment mechanism	Randomization - Implementation	Randomization - Blinding	Statistical methods	Outcomes and estimation	Limitations	Funding	Protocol	Total score
Fukuzawa et al., (2008) [12]	1	1	1	1	1	0	N/A	N/A	N/A	N/A	1	1	1	1	N/A	9
Gao et al., (2012) [13]	1	1	1	1	1	1	N/A	N/A	N/A	N/A	1	1	1	1	N/A	10
Xinlin et al., (1997) [37]	1	1	1	1	1	1	N/A	N/A	N/A	N/A	1	1	0	0	N/A	8
Lu et al., (2004) [14]	1	1	1	1	1	0	N/A	N/A	N/A	N/A	1	1	0	1	N/A	8
Lu et al., (2004) [15]	1	1	1	1	1	0	N/A	N/A	N/A	N/A	1	1	1	1	N/A	9
Oliveira et al., (2014) [16]	1	1	1	1	1	0	N/A	N/A	N/A	N/A	1	1	1	1	N/A	9
Sliva et al., (2002) [19]	1	1	1	1	0	0	N/A	N/A	N/A	N/A	0	1	0	0	N/A	5
Sliva et al., (2003) [20]	1	1	1	1	1	0	N/A	N/A	N/A	N/A	1	1	1	1	N/A	9
Song et al., (2021) [33]	1	1	1	1	0	1	N/A	N/A	N/A	N/A	1	1	1	1	N/A	9
Wang et al., (2019) [21]	1	1	1	1	1	0	N/A	N/A	N/A	N/A	1	1	1	1	N/A	9
Zhong et al., (2021) [40]	1	1	1	1	1	1	N/A	N/A	N/A	N/A	1	1	1	1	N/A	10
Zhu et al., (2000) [30]	1	1	1	1	0	0	N/A	N/A	N/A	N/A	0	1	1	0	N/A	6
Wu et al., (2012) [43]	1	1	1	1	1	1	N/A	N/A	N/A	N/A	1	1	1	1	N/A	10
Li et al., (2016) [32]	1	1	1	1	0	0	N/A	N/A	N/A	N/A	1	1	1	1	N/A	8
Chan et al., (2005) [51]	1	1	1	1	1	0	N/A	N/A	N/A	N/A	1	1	0	1	N/A	8
Chan et al., (2007) [52]	1	1	1	1	1	0	N/A	N/A	N/A	N/A	1	1	1	1	N/A	9
Hsu et al., (2012) [55]	1	1	1	1	1	1	N/A	N/A	N/A	N/A	1	1	1	1	N/A	10
Ma et al., (2008) [53]	1	1	1	1	1	1	N/A	N/A	N/A	N/A	1	1	0	0	N/A	8
Zhang et al., (2011) [50]	1	1	1	1	1	0	N/A	N/A	N/A	N/A	1	1	0	1	N/A	8
Cai et al., (2021) [65]	1	1	1	1	1	0	N/A	N/A	N/A	N/A	1	1	1	1	N/A	9
Saavedra Plazas et al., (2020) [69]	1	1	1	1	1	0	N/A	N/A	N/A	N/A	1	1	1	1	N/A	9
Nguyen and Nguyen (2015) [71]	1	1	1	1	0	0	N/A	N/A	N/A	N/A	0	1	0	0	N/A	5
Shen et al., (2019) [68]	1	1	1	1	1	0	N/A	N/A	N/A	N/A	1	1	1	1	N/A	9
Heleno et al., (2012) [70]	1	1	1	1	0	0	N/A	N/A	N/A	N/A	1	1	1	1	N/A	8
Nayak et al., (2021) [84]	1	1	1	1	1	0	N/A	N/A	N/A	N/A	1	1	1	1	N/A	9
Nayak et al., (2015) [85]	1	1	1	1	1	1	N/A	N/A	N/A	N/A	1	1	1	1	N/A	10
Nayak et al., (2010) [83]	1	1	1	1	0	0	N/A	N/A	N/A	N/A	0	1	1	0	N/A	6
Shen et al., (2020) [18]	1	1	1	1	1	1	N/A	N/A	N/A	N/A	1	1	1	1	N/A	10
Zhu et al., (2018) [86]	1	1	1	1	1	0	N/A	N/A	N/A	N/A	1	1	1	1	N/A	9
Zhu et al., (2019) [31]	1	1	1	1	1	0	N/A	N/A	N/A	N/A	1	1	1	1	N/A	9
Yang et al., (2020) [92]	1	1	1	1	1	0	N/A	N/A	N/A	N/A	1	1	1	1	N/A	9
Li et al., (2020) [79]	1	1	1	1	1	0	N/A	N/A	N/A	N/A	1	1	1	1	N/A	9
Wang et al., (2012) [17]	1	1	1	1	1	0	N/A	N/A	N/A	N/A	1	1	1	1	N/A	9
Wang et al., (2014) [82]	1	1	1	1	1	1	N/A	N/A	N/A	N/A	1	1	1	1	N/A	10
Pan et al., (2019) [81]	1	1	1	1	1	1	N/A	N/A	N/A	N/A	1	1	1	1	N/A	10
Weng et al., (2010) [100]	1	1	1	1	1	0	N/A	N/A	N/A	N/A	1	1	1	0	N/A	8
Huang et al., (2011) [95]	1	1	1	1	1	0	N/A	N/A	N/A	N/A	1	1	1	1	N/A	9
Li et al., (2013) [96]	1	1	1	1	1	0	N/A	N/A	N/A	N/A	1	1	1	1	N/A	9
Wang et al., (2013) [97]	1	1	1	1	1	0	N/A	N/A	N/A	N/A	1	1	1	1	N/A	9
Yang et al., (2016) [98]	1	1	1	1	0	0	N/A	N/A	N/A	N/A	1	1	1	1	N/A	8

Appendix 3

**Table 7:** Quality assessment of in vivo studies according to the items of the SYRCLEʼs tool

Study	1) Was the allocation sequence adequately generated and applied?	2) Were the groups similar at baseline or were they adjusted for confounders in the analysis?	3) Was the allocation to the different groups adequately concealed during?	4) Were the animals randomly housed during the experiment?	5) Were the caregivers and/or investigators blinded from knowledge which intervention each animal received during the experiment?	6) Were animals selected at random for outcome assessment?	7) Was the outcome assessor blinded?	8) Were incomplete outcome data adequately addressed?	9) Are reports of the study free of selective outcome reporting?	10) Was the study apparently free of other problems that could result in high risk of bias?
Chen et al., (2016) [41]	Unclear	Yes	Unclear	Unclear	Unclear	Unclear	Unclear	Yes	Yes	Yes
Chen et al., (2016) [36]	Unclear	Yes	Unclear	Unclear	Unclear	Unclear	Unclear	Yes	Yes	Yes
Dai et al., (2021) [44]	Unclear	Yes	Unclear	Unclear	Unclear	Unclear	Unclear	Yes	Yes	Yes
Jiao et al., (2020) [42]	Unclear	Yes	Unclear	Unclear	Unclear	Unclear	Unclear	Yes	Yes	Yes
Li et al., (2017) [35]	Unclear	Yes	Unclear	Unclear	Unclear	Unclear	Unclear	Yes	Yes	Yes
Na et al., (2017) [26]	Unclear	Yes	Unclear	Unclear	Unclear	Unclear	Unclear	Yes	Yes	Yes
Shi et al., (2021) [39]	Unclear	Yes	Unclear	Unclear	Unclear	Unclear	Unclear	Yes	Yes	Yes
Su et al., (2018) [23]	Unclear	Yes	Unclear	Unclear	Unclear	Unclear	Unclear	Yes	Yes	Yes
Su et al., (2018) [28]	Unclear	Yes	Unclear	Unclear	Unclear	Unclear	Unclear	Yes	Yes	Yes
Zhang et al., (2019) [25]	Unclear	Yes	Unclear	Unclear	Unclear	Unclear	Unclear	Yes	Yes	Yes
Pan et al., (2019) [27]	Unclear	Yes	Unclear	Unclear	Unclear	Unclear	Unclear	Yes	Yes	Yes
Wang et al., (2012) [29]	Unclear	Yes	Unclear	Unclear	Unclear	Unclear	Unclear	Yes	Yes	Yes
He et al., (2020) [24]	Unclear	Yes	Unclear	Unclear	Unclear	Unclear	Unclear	Yes	Yes	Yes
Guo et al., (2009) [54]	Unclear	Yes	Unclear	Unclear	Unclear	Unclear	Unclear	Yes	Yes	Yes
Yue et al., (2008) [38]	Unclear	Yes	Unclear	Unclear	Unclear	Unclear	Unclear	Yes	Yes	Yes
Bao et al., (2002) [48]	Unclear	Yes	Unclear	Unclear	Unclear	Unclear	Unclear	Yes	Yes	Yes
Bao et al., (2001) [49]	Unclear	Yes	Unclear	Unclear	Unclear	Unclear	Unclear	Yes	Yes	Yes
Dai et al., (2019) [75]	Unclear	Yes	Unclear	Unclear	Unclear	Unclear	Unclear	Yes	Yes	Yes
Fu et al., (2019) [34]	Unclear	Yes	Unclear	Unclear	Unclear	Unclear	Unclear	Yes	Yes	Yes
Liu et al., (2002) [22]	Unclear	Yes	Unclear	Unclear	Unclear	Unclear	Unclear	Yes	Yes	Yes
Bao et al., (2001) [56]	Unclear	Yes	Unclear	Unclear	Unclear	Unclear	Unclear	Yes	Yes	Yes
Bao et al., (2001) [57]	Unclear	Yes	Unclear	Unclear	Unclear	Unclear	Unclear	Yes	Yes	Yes
Li et al., (2020) [61]	Unclear	Yes	Unclear	Unclear	Unclear	Unclear	Unclear	Yes	Yes	Yes
Liu et al., (2021) [59]	Unclear	Yes	Unclear	Unclear	Unclear	Unclear	Unclear	Yes	Yes	Yes
Su et al., (2021) [58]	Unclear	Yes	Unclear	Unclear	Unclear	Unclear	Unclear	Yes	Yes	Yes
Wang et al., (2017) [62]	Unclear	Yes	Unclear	Unclear	Unclear	Unclear	Unclear	Yes	Yes	Yes
Wu et al., (2020) [60]	Unclear	Yes	Unclear	Unclear	Unclear	Unclear	Unclear	Yes	Yes	Yes
Ma et al., (2009) [63]	Unclear	Yes	Unclear	Unclear	Unclear	Unclear	Unclear	Yes	Yes	Yes
Sang et al., (2021) [66]	Unclear	Yes	Unclear	Unclear	Unclear	Unclear	Unclear	Yes	Yes	Yes
Levin et al., (2017) [72]	Unclear	Yes	Unclear	Unclear	Unclear	Unclear	Unclear	Yes	Yes	Yes
Zhang et al., (2021) [73]	Unclear	Yes	Unclear	Unclear	Unclear	Unclear	Unclear	Yes	Yes	Yes
Zhan et al., (2016) [87]	Unclear	Yes	Unclear	Unclear	Unclear	Unclear	Unclear	Yes	Yes	Yes
Jiang et al., (2021) [88]	Unclear	Yes	Unclear	Unclear	Unclear	Unclear	Unclear	Yes	Yes	Yes
Lai et al., (2020) [91]	Unclear	Yes	Unclear	Unclear	Unclear	Unclear	Unclear	Yes	Yes	Yes
Shaher et al., (2020) [89]	Unclear	Yes	Unclear	Unclear	Unclear	Unclear	Unclear	Yes	Yes	Yes
Wang et al., (2015) [90]	Unclear	Yes	Unclear	Unclear	Unclear	Unclear	Unclear	Yes	Yes	Yes
Gao et al., (2010) [74]	Unclear	Yes	Unclear	Unclear	Unclear	Unclear	Unclear	Yes	Yes	Yes
Jin et al., (2013) [78]	Unclear	Yes	Unclear	Unclear	Unclear	Unclear	Unclear	Yes	Yes	Yes
Liu et al., (2021) [76]	Unclear	Yes	Unclear	Unclear	Unclear	Unclear	Unclear	Yes	Yes	Yes
Xie et al., (2016) [77]	Unclear	Yes	Unclear	Unclear	Unclear	Unclear	Unclear	Yes	Yes	Yes
Zhou et al., (2012) [80]	Unclear	Yes	Unclear	Unclear	Unclear	Unclear	Unclear	Yes	Yes	Yes
Zhao et al., (2021) [93]	Unclear	Yes	Unclear	Unclear	Unclear	Unclear	Unclear	Yes	Yes	Yes
Jiao et al., (2020) [94]	Unclear	Yes	Unclear	Unclear	Unclear	Unclear	Unclear	Yes	Yes	Yes
Ge et al., (2009) [67]	Unclear	Yes	Unclear	Unclear	Unclear	Unclear	Unclear	Yes	Yes	Yes

Appendix 4

**Table 8:** Quality assessment of retrospective study using the Study Quality Assessment Tools (SQAT) Question 1. Was the research question or objective in this paper clearly stated? Question 2. Was the study population clearly specified and defined? Question 3. Was the participation rate of eligible persons at least 50%? Question 4. Were all the subjects selected or recruited from the same or similar populations (including the same time periods)? Were inclusion and exclusion criteria for being in the study prespecified and applied uniformly to all participants? Question 5. Was a sample size justification, power description, or variance and effect estimates provided? Question 6. For the analyses in this paper, were the exposure(s) of interest measured prior to the outcome(s) being measured? Question 7. Was the timeframe sufficient so that one could reasonably expect to see an association between exposure and outcome if it existed? Question 8. For exposures that can vary in amount or level, did the study examine different levels of the exposure as related to the outcome (e.g., categories of exposure, or exposure measured as continuous variable)? Question 9. Were the exposure measures (independent variables) clearly defined, valid, reliable, and implemented consistently across all study participants? Question 10. Was the exposure(s) assessed more than once over time? Question 11. Were the outcome measures (dependent variables) clearly defined, valid, reliable, and implemented consistently across all study participants? Question 12. Were the outcome assessors blinded to the exposure status of participants? Question 13. Was loss to follow-up after baseline 20% or less? Question 14. Were key potential confounding variables measured and adjusted statistically for their impact on the relationship between exposure(s) and outcome(s)?

Article	Question														Overall
	1	2	3	4	5	6	7	8	9	10	11	12	13	14	
Wang et al., (2018) [98]	1	1	1	1	0	1	NA	0	1	0	1	0	1	1	Fair

Appendix 5

**Table 9:** Quality assessment of case reports using the Study Quality Assessment Tools (SQAT) 1. Was the study question or objective clearly stated? 2. Was the study population clearly and fully described, including a case definition? 3. Were the cases consecutive? 4. Were the subjects comparable? 5. Was the intervention clearly described? 6. Were the outcome measures clearly defined, valid, reliable, and implemented consistently across all study participants? 7. Was the length of follow-up adequate? 8. Were the statistical methods well-described? 9. Were the results well-described?

Article	Question									Overall
	1	2	3	4	5	6	7	8	9	
Liang et al., 2013 [99]	1	1	1	1	1	1	1	0	1	Good
Yan et al., 2014 [45]	1	1	0	1	1	1	1	0	1	Fair

Appendix 6

**Table 10:** Quality assessment for RCT using ROB2 from Cochrane RCT: randomized control trial; ROB2: risk-of-bias tool for randomized trials

Study	Domain 1: Risk of bias arising from the randomization process	Domain 2: Risk of bias due to deviations from the intended interventions (effect of assignment to intervention)	Domain 2: Risk of bias due to deviations from the intended interventions (effect of adhering to intervention)	Domain 3: Missing outcome data	Domain 4: Risk of bias in measurement of the outcome	Domain 5: Risk of bias in selection of the reported result	Domain 6: Overall bias
Suprasert et al., (2013) [45]	Low	Low	Some concerns	Low	Low	Low	Some concerns (moderate risk of bias)

Appendix 7

**Table 11:** Quality assessment for non-RCT using ROB2 from Cochrane RCT: randomized control trial; ROB2: risk-of-bias tool for randomized trials

Study	1. Bias due to confounding	2. Bias in selection of participants into the study	3. Bias in classification of interventions	4. Bias due to deviations from intended interventions	5. Bias due to missing data	6. Bias in measurement of outcomes	7. Bias in selection of the reported result	8. Overall bias
Deng et al., (2021) [64]	Low	Low	Low	Low	Low	Moderate	Moderate	Moderate
Zhao et al., (2012) [47]	Low	Low	Low	Low	Low	Moderate	Moderate	Moderate
